# Enumerating viable phytoplankton using a culture-based Most Probable Number assay following ultraviolet-C treatment

**DOI:** 10.1007/s10811-017-1254-8

**Published:** 2017-09-25

**Authors:** Hugh L. MacIntyre, John J. Cullen, Trina J. Whitsitt, Brian Petri

**Affiliations:** 1 Department of Oceanography, Dalhousie University, PO Box 15000, Halifax, NS B3H 4R2 Canada; 2 Trojan Technologies, 3020 Gore Rd, London, ON N5V 4T7 Canada

**Keywords:** UVC treatment, Phytoplankton, Ballast water, Stress, Viability, Delayed repair

## Abstract

Ballast water management systems (BWMS) must be tested to assess their compliance with standards for the discharge of organisms, for example in the ≥ 10- and < 50-μm size category, which is dominated by phytoplankton. Assessment of BWMS performance with the vital stains fluorescein diacetate + 5-chlorofluorescein diacetate, required by regulations in the USA, is problematic in the case of ultraviolet-C (UVC) radiation. This is because UVC targets nucleotides—and thus reproduction, hence viability—rather than membrane integrity, which is assayed by the stains. The Serial Dilution Culture-Most Probable Number (SDC-MPN) method, long used to enumerate fragile phytoplankton from natural communities, is appropriate for counting viable phytoplankton. We developed QA/QC “best practice” criteria for its application as a robust and repeatable assay of viable cells in cultures of phytoplankton before and after experimental treatment, then constructed dose-response curves for UVC-induced loss of viable cells in 12 species of phytoplankton from seven divisions. Sensitivity to UVC, expressed as the dose required to reduce viability by 99%—the criterion for type approval of treatment systems—varied more than 10-fold and was not correlated with cell size. The form of the dose-response curves varied between taxa, with most having a threshold dose below which there was no reduction in viability. Analysis of the patterns of growth indicates that if recovery from treatment occurred, it was complete in 1 or 2 days in > 80% of cases, long before the assays were terminated. We conclude that the SDC-MPN assay as described is robust and adaptable for use on natural phytoplankton.

## Introduction

Harmful algal blooms (HABs) can be costly in both economic and ecological terms. There is much debate over the conditions that give rise to them (e.g., Davidson et al. 2012; Glibert et al. 2012), but one contributory factor is fundamental: viable cells of the bloom-forming organism must be present for a bloom to develop. There is a significant potential for transport and introduction of HAB taxa through ships’ ballast water (e.g., Doblin et al. 2004; Bolch and de Salas 2007; Roy et al. 2012), which was one stimulus for the International Maritime Organization (IMO) adopting the *International Convention for the Control and Management of Ships’ Ballast Water and Sediments* at a Diplomatic Conference in London in 2004 (IMO 2004). The treaty was ratified by the requisite number of signatory nations in September 2016, meaning that ballast water treatment will be subject to international law one year later. National regulation is already in effect in the USA, governed by the *Nonindigenous Aquatic Nuisance Prevention and Control Act* (1990) and the *National Invasive Species Act* (1996). Regulatory authority resides with the United States Coast Guard (USCG), which entered into a formal Memorandum of Understanding with the US Environmental Protection Agency to develop procedures for evaluating ballast water management systems (BWMS).

The regulations governing ballast water management address many categories of potentially invasive organisms. Two size classes of plankton are regulated: organisms that are ≥ 10 to < 50 μm in minimum dimension and those that are ≥ 50 μm in minimum dimension. Potential treatment methods include filtration, ultraviolet irradiation, heating, ultrasound, magnetic separation, electrolysis, chlorination, ozonation, and use of other chemical biocides (reviewed by Tsolaki and Diamadopoulos 2010). Germicidal UV (ultraviolet-C, UVC) radiation is a mature technology that is used extensively in disinfection of wastewater, drinking water, and recycled water and which was demonstrated to be an effective disinfectant for marine microbes more than a century ago (Henri et al. 1910) and to be effective with an alga more than 60 years ago (Redford and Myers 1951).

To be granted type approval, a BWMS must meet performance standards as demonstrated by a third-party testing facility to the satisfaction of the responsible administration. The standard for the 10–50-μm size range is fewer than 10 viable (IMO 2008) or living (USCG 2012) organisms mL^−1^, reduced from untreated concentrations of at least 1000 organisms mL^−1^, corresponding to a 99% reduction in viable or living organisms. The path to type approval therefore requires not only the development of effective treatment technologies but also reliable assays that can verify performance metrics. In this study, we use a 99% reduction as a metric to evaluate UVC treatment, recognizing that it is relevant for type approval of BWMS. It is important to recognize that this is different from doses that would be applied to meet regulatory compliance. Because a BWMS must treat to the regulatory standard of 10 viable/living cells mL^−1^, the applied doses might be higher or lower depending on the initial concentrations of cells in the 10–50-μm size range.

The effectiveness of UVC is described using dose-response relationships (e.g., Hijnen et al. 2006; Haji Malayeri et al. 2016). The primary targets of UVC radiation are nucleotides (Sinha and Hader 2002; Cadet et al. 2014), and in the case of the phytoplankton, their dimerization can impair a cell’s ability to reproduce at doses that have comparatively minor effects on secondary targets such as photosynthesis (e.g., Redford and Myers 1951). Consequently, a definitive assay would be a test of the cells’ ability to reproduce (Breeuwer and Abee 2000), measured in the relative reduction in numbers of viable cells as a function of UVC exposure. However, there are very few published dose-response relationships for phytoplankton that could be used to develop appropriate BWMS, although manufacturers undoubtedly have conducted their own tests. Calculations on data for the chlorophytes *Tetraselmis suecica* and *Chlamydomonas reinhardtii* (Chaudhari et al. 2015; Olsen et al. 2015) suggest that a dose of 300–490 mJ cm^−2^ would be required to effect the minimum 99% reduction of viable cell numbers required to meet the test standard (for phytoplankton in this context, “cell” is equivalent to a regulated “organism”). The data are not truly comparable as the former used medium-pressure lamps (broad-spectrum UV) while the latter used low-pressure lamps (narrow-spectrum UVC). Even so, this is very high compared to the range of doses that give 99% disinfection in viruses, bacteria, and freshwater protozoa such as *Cryptosporidium* and *Giardia* and that have been used to design systems for treatment of drinking water (4–111 mJ cm^−2^ in data compiled by Hijnen et al. 2006; Haji Malayeri et al. 2016). In contrast, field experiments with mixed assemblages suggest that phytoplankton might be less resistant than bacteria (First and Drake 2014). There is, therefore, a need to quantify dose-response curves for viable phytoplankton cells to assess how effective UVC is as a treatment technology.

Reliable enumerations of viable organisms are also needed for assessing the effectiveness of BWMS. Although testing based on SDC-MPN assays for viable cells (see Wright and Welschmeyer 2015) has been explicitly rejected by the US Coast Guard for type approval of UVC BWMS, the primary reason was because tests of viability do not use the criterion for counting living organisms, as required by US regulations (https://www.uscg.mil/foia/docs/FINAL-AG.pdf). Notably, BWMS type approvals have been granted by other administrations based on SDC-MPN assays (IMO 2017), and SDC-MPN is explicitly recognized as the appropriate assay for testing UVC-treated drinking water in the USA (USEPA 2006).

Discharge standards expressed in living or viable organisms mL^−1^ imply that assessment of BWMS performance requires classification and enumeration of individual organisms (for phytoplankton, cells) rather than a bulk measurement of vitality or viability (Zetsche and Meysman 2012). Even so, there is significant value in “indicative” bulk parameters, such as variable fluorescence (Wright et al. 2015; Bradie et al. 2017; First et al. 2017), for the rapid assessment of BWMS performance, with the recognition that such assays must ultimately be related to corresponding organism counts and status (i.e., viable or living). The need for enumeration of viable phytoplankton cells stands. While “regrowth” experiments—incubation of treated samples under conditions favorable for growth (e.g., Liebich et al. 2012; Stehouwer et al. 2015; Wright et al. 2015)—can demonstrate unequivocally the persistence of viable cells, they do not translate to the concentrations of viable cells remaining after treatment. However, quantitative estimates can be made by adaptation of a venerable technique used in microbiology, SDC-MPN (McCrady 1915; Cochran 1950). The assay is based on testing for the presence of viable cells in serial dilutions of a sample by monitoring for growth. As dilution increases toward infinity and the probability of a single viable cell remaining in a diluted sample declines to 0, so does the probability of detecting growth in the sample. The most probable number of organisms in the undiluted sample is determined statistically from the number of tubes in a replicated dilution series in which there is evidence of growth (Cochran 1950; Throndsen 1978).

The SDC-MPN method has been used in several contexts in studies of microalgae and cyanobacteria. It has been used to enumerate cyanobacteria and diatom, raphidophyte, and dinoflagellate propagules from soils and sediments (Imai et al. 1986; Othman and Wollum 1987; An et al. 1992; Itakura et al. 1997; Harris et al. 1998; McQuoid 2002) and to estimate the abundances of difficult-to-preserve subpopulations within natural communities of phytoplankton (Knight-Jones 1951; Throndsen 1978; Furuya and Marumo 1983; Jochem 1990; Throndsen and Kristiansen 1991; Backe-Hansen and Throndsen 2002; Andersen and Throndsen 2003; Cerino and Zingone 2006; reviewed recently by Cullen and MacIntyre 2016). More recently, it has been used to estimate viability after UVC treatment (Oemcke et al. 2004; First and Drake 2014; Casas-Monroy et al. 2016; Chaudhari et al. 2015; Wright and Welschmeyer 2015).

Although the SDC-MPN method is a benchmark test in microbiology, against which other methods of enumerating bacteria can be assessed (e.g., Russell 2000), and although it has been used to enumerate phytoplankton for more than six decades, its applicability for enumerating total viable phytoplankton in a regulatory framework will require further validation, including “best practice” procedures that have yet to be fully developed. In particular, as addressed by Cullen and MacIntyre (2016), it is critical to assess whether the lack of observed growth in an assay tube is a confirmed absence of viable cells, or a false-negative error arising because the assay was not conducted for long enough. This primary uncertainty with the use of SDC-MPN on natural phytoplankton—the assumption that a species will grow under the assay conditions—is obviated when using lab cultures that are diluted and then incubated in growth conditions that are known to support vigorous growth. Experiments on cultures thereby facilitate optimization, replication, and interpretation of results.

In this study, we have developed rigorous “best practice” criteria for laboratory experiments using the SDC-MPN method and have quantified repeatable UVC dose-response relationships on cultures of phytoplankton from seven divisions. Although the qualitative and quantitative responses differed between taxa, replicated experiments assessed with SDC-MPN showed that UVC was an effective disinfectant in all cases. We have also tested for the likelihood that the assay is biased by undetected delayed recovery and conclude that it is not an issue. Because the experiments were conducted on cells in a reproducible growth condition, the dose-response curves could be used to ground-truth other viability assays without the need to repeat the experiments, which involved monitoring > 3000 individual cultures over 2 ½ years.

## Materials and methods

### Culturing and chlorophyll *a* fluorescence

Cultures of phytoplankton were obtained from the National Center for Marine Algae and Microbiota (NCMA, East Boothbay, ME, USA). The isolates were grown at 18 ± 0.3 °C on a 12:12 L:D photocycle at 130 ± 5 μmol photons m^−2^ s^−1^ of PAR, provided by Osram FL40SS-W/37 fluorescent bulbs. Cultures were maintained at low optical density in 40-mL volumes in balanced, semicontinuous, nutrient-replete growth (MacIntyre and Cullen 2005; Wood et al. 2005) by inoculation into fresh medium approximately once per generation (1–4 days, Table 1). The cultures were grown on f/2 (Guillard 1975) or L1 (Guillard and Hargraves 1993) seawater media. The growth media were prepared from tangential-flow-filtered coastal seawater (salinity c. 30) that was enriched with nutrients and delivered into autoclaved glassware through a 0.2-μm Whatman Polycap AS disposable filter capsule. Culture transfers were performed in a laminar flow hood that was wiped down with methanol prior to use. All tubes were flamed on opening and on closure to prevent contamination. All vessels used in culturing were cleaned prior to use by soaking in 5% Micro-90 cleaning solution (Z281506, Sigma Aldrich) and then in 2% HCl, rinsing copiously with E-Pure water (Barnstead Nanopure/APS Water Services Corporation, Lake Balboa, CA, USA) after each cleaning step. They were then sterilized in an autoclave.

**Table 1:** Cultures used in the study. Strain numbers (CCMP) are from the National Center for Marine Algae. Cultures were grown on f/2 or L1 seawater media, as indicated. Average cell diameter (ACD) was determined by a microscope. Generation time (*t*_gen_) is calculated from the between-day specific growth rate during balanced growth (mean ± standard deviation, 100 < *n* < 450)

Division	Species	Growth medium	ACD (μm)	*t*_gen_ (days)
Cyanophyta	*Synechococcus elongatus* (CCMP1630)	f/2	N/A	1.10 ± 0.10
Chlorophyta	*Chlamydomonas_*cf. sp. (CCMP1268)	f/2	12.4 ± 1.7	1.69 ± 0.37
	*Pyramimonas parkeae* (CCMP725)	L1	13.1 ± 1.5	1.20 ± 0.10
Euglenophyta	*Eutreptiella* cf. *gymnastica* (CCMP1594)	L1	23.8 ± 4.3	1.69 ± 0.29
Cryptophyta	*Rhodomonas salina* (CCMP1319)	L1	11.6 ± 1.6	1.08 ± 0.14
Haptophyta	*Isochrysis galbana* (CCMP1323)	L1	5.2 ± 0.6	1.03 ± 0.06
	*Phaeocystis globosa* (CCMP627)	L1	7.7 ± 1.1	0.86 ± 0.08
Heterokontophyta	*Heterosigma akashiwo* (CCMP1912)	L1	15.5 ± 2.8	1.24 ± 0.16
	*Thalassiosira weissflogii* (CCMP1050)	f/2	13.0 ± 1.4	0.84 ± 0.05
Dinophyta	*Alexandrium andersoni* (CCMP3376)	L1	23.3 ± 1.6	2.24 ± 0.36
	*Amphidinium carterae* (CCMP1314)	f/2	13.4 ± 1.5	1.31 ± 0.15
	*Lingulodinium polyedra* (CCMP1738)	L1	43.3 ± 4.3	3.85 ± 1.71
	*Scrippsiella trochoidea* (CCMP2775)	L1	20.3 ± 3.7	1.69 ± 0.33

*N/A*: not measured

The cultures were grown in 50-mL borosilicate glass tubes with HDPE screw-caps that could be inserted while still sealed into the interrogation volumes of two fluorometers, so that fluorescence could be measured without compromising sterility (Brand et al. 1981). Specific fluorescence-based growth rates (*μ*_*F*_, day^−1^; Table 2) were calculated daily (Wood et al. 2005) from the change in dark-acclimated and dilution-corrected Chl*a* fluorescence (Eq. 1, Table 3). Fluorescence was measured daily with a Turner 10AU fluorometer (Turner Designs, USA) 3–5 h after the start of the night phase of the L:D cycle. Variable Chl*a* fluorescence was also measured daily with a FIRe fluorometer (Satlantic, Canada). Both fluorometers were blanked with filtered seawater and the FIRe was standardized daily with a 100 μmol L−^1^ solution of rhodamine *b* (R6625, Sigma Aldrich) in E-Pure water. Estimates of the minimum, maximum, and variable fluorescence (*F*_0_, *F*_m_, *F*_v_; Table 2) were extracted from the single-turnover flash induction curves using Fireworx software (Audrey Barnett, http://sourceforge.net/projects/fireworx/). Curve fits were performed with the curvature parameter in the fit, *p*, set to 0.

**Table 2:** Definitions of terms

Parameter	Definition	Unit
ACD	Average cell diameter, = 2 ⋅ √(Cross-sectional area/π)	μm
CF(*i*)	Concentration factor in tier *i* of SDC-MPN assay	Dimensionless
CV[*x*]	Coefficient of variation in parameter *x*	%
*d*	Dilution (proportion of culture replaced with fresh medium)	Dimensionless
*D*	UVC dose	mJ cm^−2^
*D*[0.01]	UVC dose required to reduce RV to 10^−2^	mJ cm^−2^
*D*_Th_	Threshold dose of UVC below which there is no reduction in viability	mJ cm^−2^
*D*′	UVC dose normalized to *D*[0.01]in *Phaeocystis*	Relative
*D*′[0.01], ${D}_{\mathrm{Th}}^{\prime }$	UVC dose required to reduce RV to 10^−2^ and threshold dose, normalized to *D*[0.01] for *Phaeocystis*	Relative
DR	Dilution ratio between tiers of SDC-MPN assay	Dimensionless
*F*	Dark-acclimated Chl*a* fluorescence in vivo	Arb.
*F*′	*F* normalized to *N*_viable_	Dimensionless
*F*_0_, *F*_m_	Minimum and maximum dark-acclimated Chl*a* fluorescence in vivo	Arb.
*F*_Init_	Intercept for linear regression of ln(*F*_0_) on *t* in dilution 10^*X*^ of a dilution series 10^*X*^, 10^*X*−1^, and 10^*X*−2^	Arb.
${F}_{\mathrm{Init}}^{\prime }$	*F*_Init_ normalized to *N*_viable_	Dimensionless
*F*_v_	Variable dark-acclimated Chl*a* fluorescence in vivo, = *F*_m_ *− F*_0_	Arb.
*F*_v_ */*F_m_	Ratio of variable to maximum dark-acclimated Chl*a* fluorescence in vivo	Dimensionless
*k*, *k*_1_, *k*_2_	Sensitivity coefficients for decline of viability with UVC dose	mJ^−1^ cm^2^
*k*′	Sensitivity coefficient scaled to the UVC dose required to effect a 10^−2^ reduction in viability in *Phaeocystis*	Relative
LLD_Cell_	Lower limit of detection obtained by linear regression of *F* on *N*	Cells mL^−1^
LLD_*F*_	Sensor output (fluorescence) at the lower limit of detection	Arb.
*m*	Slope from a linear regression of *F* on *N*	Arb.
MPN	Most probable number of viable cells	Cells
MPN_med_	Median of tabulated value of MPN for calculable tube scores: 22 for 3- and 5-replicate assays, 21 for a 10-replicate assay (FDA 2010)	Cells
*N*	Cell concentration	Cells mL^−1^
*N*_0_	Concentration of cells in a culture prior to treatment	Cells mL^−1^
*N*_viable_	Concentration of viable cells in a treated or untreated culture	Cells mL^−1^
*q*	Number of tiers in SDC-MPN assay	Dimensionless
*r*	Number of tubes in each tier of SDC-MPN assay	Dimensionless
*R* ^2^[CumFl]	Coefficient of determination for linear regression of ln*-*transformed, dilution-corrected *F* on *t*	Dimensionless
RV	Relative viability, = *N*_viable_/*N*_0_	Dimensionless
RV_*D*_	Relative viability after application of UVC dose *D* before and after dark hold	Dimensionless
RV_*D* = 0_	Relative viability of a sample held in darkness without UVC treatment	Dimensionless
*t*	Time	day
*T*_0_	Untreated parent sample	
*t*_end_	Temporal end point for an MPN assay	day
*t*_gen_	Generation time, = ln(2)/*μ*_*F*_	day
*V*	Volume of sample plus medium cultured in MPN assays	mL
*X*	Integer defining the range of dilutions used in the MPN assay: 10^*X*^, 10^*X*−1^, and 10^*X*−2^	Dimensionless
*α*	Partitioning coefficient for relative weight of two UVC targets at a given UVC dose	Dimensionless
*μ*_*F*_	Fluorescence-based specific growth rate, the slope in a regression of ln(*F*) on *t*	day^−1^
*σ*_*x*/*y*_	Standard error of the *y* estimate in a linear regression of *F* on *N*	Arb.

**Table 3:** Summary of QA/QC measures used to grow cultures in balanced growth and estimate the concentration of viable cells by the SDC-MPN method. Equation 3 is from Anderson (1989)

Procedure	Condition	Specification	Eq. number
Culturing	Determination of specific growth rate	${\mu}_F=\frac{1}{t_2-{t}_1}\cdot \ln \left(\frac{F_2\cdot {\left[1-d\right]}^{-1}}{F_1}\right)$	1
	Definition of balanced growth		
	For *μ*_*F*_ ≥ 0.5 day^−1^:	CV[*μ*_*F*_] ≤ 10% and CV[*F*_v_/*F*_m_] ≤ 10% for 10 ⋅ *t*_gen_	2a
	For *μ*_*F*_ < 0.5 day^−1^:	*R* ^2^[CumFl] ≥ 0.995 and CV[*F*_v_/*F*_m_] ≤ 10% for 10 ⋅ *t*_gen_	2b
Instrument assessment	Determination of lower limit of detection	${\mathrm{LLD}}_{\mathrm{Cell}}=\frac{3{\upsigma}_{x/y}}{m}$ where ${\sigma}_{x/y}=\sqrt{\left[\frac{\sum_i{\left({F}_{\mathrm{obs}}-{F}_{\mathrm{regr}}\right)}^2}{n-2}\right]}$	3
MPN assay	Determination of appropriate dilution range	10^*X*^, 10^*X* − 1^, and 10^*X* − 2^ where $X=\left\lfloor \log, \left(\frac{{\mathrm{MPN}}_{\mathrm{med}}}{N_{\mathrm{viable}}\cdot V}\right)\right\rceil -1$	4
	Calculating the concentration of viable cells	*N*_viable_ = MPN ⋅ *V* ^−1^ ⋅ 10^−(1 − *X*)^	5
	Time to complete test for a dilution series	${t}_{\mathrm{end}}=\left(Y\cdot \ln (2)-\ln \left(\frac{10^{X-x}\cdot {F}_{\mathrm{Init}}}{{\mathrm{LLD}}_F}\right)\right)\cdot {\mu}_F^{-1}$ or	6a
		${t}_{\mathrm{end}}=\left(Y\bullet \ln (2)-\ln \left(\frac{10^{X-x}\cdot {N}_{\mathrm{viable}}}{{\mathrm{LLD}}_{\mathrm{Cell}}}\right)\right)\cdot {\mu}_F^{-1}$ where *Y* is either 5 or 3 (see text) and *x = X*, *X* − 1, or *X* − 2 for the dilution series 10^*X*^, 10^*X* − 1^, and 10^*X* − 2^	6b
	Scoring tests		
	Positive scores:	*F* ≥ 8 ⋅ max(LLD_*F*_, *F*_start_) at *t ≤ t*_end_	7a
	Negative scores:	*F* < 8 ⋅ max(LLD_*F*_, *F*_start_) at *t = t*_end_	7b

An essential element of the experimental approach was maintenance of the cultures in balanced growth using semicontinuous culture (see MacIntyre and Cullen 2005), thereby avoiding the variations of physiological status and life stage that are inherent during unbalanced growth (Harrison et al. 1990; Han et al. 2002; Guan and Lu 2010), e.g., during typical batch-culture experiments. The requirement for balanced growth ensures that all cells are actively growing and fully acclimated to defined growth conditions. Under these conditions, the cells are demonstrably all viable (MacIntyre and Cullen 2016), which is the appropriate starting point for tests of a stressor. Stock cultures were judged to be in balanced growth when the coefficients of variation (CV) for both daily growth rate and *F*_v_/*F*_m_ were < 10% over 10 generations (Eq. 2, Table 3) for cultures for which *μ*_*F*_ was ≥ 0.5 day^−1^. Cultures in which *μ*_*F*_ was < 0.5 day^−1^ were judged to be in balanced growth when the coefficient of determination of a linear regression of ln-transformed, dilution-corrected fluorescence on time was > 0.995 and the CV for *F*_v_/*F*_m_ was < 10% over 10 generations (Eq. 2, Table 3). The second set of criteria were adopted because stochastic error in estimating fluorescence effectively precluded meeting the criterion for < 10% variation in *μ*_*F*_ at low growth rates.

When a culture had achieved balanced growth, an inoculum of 15–25 mL was transferred into a sterile 750-mL polystyrene tissue-culture flask. Its total volume was increased by adding fresh medium daily to maintain constant fluorescence at that time in the photoperiod until the volume required for experimentation was reached. The experimental cultures were monitored daily using the same protocols as for the stock lines to confirm that the test material remained in balanced growth. The only difference was that subsamples were decanted into sterile 50-mL culture tubes in the laminar flow hood and dark-acclimated prior to measuring fluorescence. After measurement, the subsample was returned to the culture flask under sterile conditions in the hood.

### Limit of detection and average cell diameter

The fluorometer’s lower limit of detection (LLD_Cell_; Eq. 3, Table 3), the lowest cell concentration that could be distinguished from a blank, was measured for each species by regression (Anderson 1989; Miller and Miller 2005). Dark-acclimated fluorescence was measured on aliquots of steady-state cultures that had been diluted to 20, 40, 60, 80, and 100% of the initial concentration with fresh medium. Cell concentration in each steady-state culture (*N*_0_, cells mL^−1^; Table 2) was determined with an Accuri C6 flow cytometer (Becton Dickinson, Canada). Counts were gated on Chl*a* fluorescence (emission > 670 nm) at a level above the signals generated from 0.2-μm-filtered E-Pure water, to eliminate the contribution of noise and particles other than the phytoplankton. Fluorescence in the diluted samples was then regressed on cell concentration, obtained from *N*_0_ and % dilution, and LLD_Cell_ calculated from the standard error of the fluorescence estimates and the slope of the regression (Eq. 3, Table 3). The corresponding fluorescence intensity, LLD_*F*_ (Arb.), was determined from LLD_Cell_ using the coefficients of the regression.

Average cell diameter (ACD; Tables 1 and 2) was calculated from cross-sectional area, which was measured on a minimum of 50 cells per culture, using an AX10 microscope with AxioCam ERc5s camera (Zeiss, Germany). Cell areas were analyzed in ZEN software, calibrated using the lines on a Reichert Bright-Line hemocytometer (Hausser Scientific, USA).

### UV treatment

Cultures were treated with defined doses of UVC radiation using a custom-built, low-pressure collimated beam (Bolton and Linden 2003). The output of the collimated beam (mW cm^−2^) was measured before each use with a NIST-traceable ILT1700 radiometer (International Light Technologies, USA), which was calibrated annually. The dose (mJ cm^−2^) was applied by setting an appropriate time of exposure for 50 mL of culture stirred in a glass reaction vessel 5 cm in diameter and 2.5 cm in depth. The doses applied were all within the range of doses applied in municipal water treatment (USEPA 2006; NWRI 2012). The appropriate time of exposure for each dose was calculated from the average intensity (mW cm^−2^) in the reaction vessel, calculated from the measured intensity at the sample surface and corrected for the reflection, Petri, water, and divergence factors (Bolton and Linden 2003). Transmittance was measured in a 1-cm quartz cuvette with a P100 UV254 meter (Real Tech Inc., Canada). Transmittance at 254 nm in the cultures was 81 ± 7% (mean ± standard deviation; *n* = 136) for independent samples.

The experimental regimen was designed to mimic that of a UV-based treatment system undergoing land-based testing for IMO approval: the IMO G8 protocol (IMO 2008) specified that such testing must include a minimum dark hold of 5 days between uptake and discharge. Accordingly, dose-response curves were based on division of a culture into 50-mL aliquots, irradiation of each, incubation in darkness for 5 days, and a second irradiation at the same dose. Where reported, the UV dose refers to the dose applied in each treatment, so is equivalent to half of the total dosage received by each sample. The culture tubes were maintained in darkness by shrouding them in four thicknesses of aluminum foil in the incubator. The following terms are used to distinguish between treatments: *T*_0_ samples are samples in balanced growth that have received no further manipulation; treated samples were either irradiated and maintained in darkness for 5 days as described above (UV-treated), or were held in darkness for 5 days but without UV irradiation (dark controls).

### SDC-MPN growth assays

Cell viability was determined using the serial dilution culture method with MPN (McCrady 1915; Cochran 1950). Its application for phytoplankton (Throndsen 1978) was modified by using fluorescence rather than microscopy to monitor growth (cf. First and Drake 2014). The SDC-MPN assay has a quantitative range of detection of 1.8–1600 cells when conducted with 5 replicate tubes at dilutions of 10^−1^, 10^−2^, and 10^−3^ (FDA 2010). The combination of the dynamic range of the assay and the assay volumes gives the detectable range of viable cells (e.g., 0.36–320 cells mL^−1^ for 5-mL assay volumes). If the concentration of viable cells in the parent sample, *N*_viable_, exceeds the detectable range, more dilution is required. So, to maximize compatibility between the dilutions and estimated *N*_viable_, dilution ranges were set as 10^*X*^, 10^*X*−1^, and 10^*X*−2^, in which *X* is an integer based on the estimated number of viable cells and the median tabulated value of MPN (Eq. 4, Table 3). The estimate of viable cells came from either (i) total cell concentration, *N*_0_ (cells mL^−1^, determined by flow cytometry), in untreated *T*_0_ samples (demonstrably equivalent to *N*_viable_, see “Results” and MacIntyre and Cullen 2016); or (ii) from estimated survival of viable cells after treatment, obtained from *N*_0_ and preliminary range-finding tests on UVC-treated samples. For the range of *N*_viable_ assayed (3.6 × 10^−1^ to 1.8 × 10^5^ cells mL^−1^), *X* ranged from 0 to − 6.

Test samples were serially diluted with fresh, sterile growth media to 10^*X*^, 10^*X* − 1^, and 10^*X* − 2^, calculated according to Eq. 4 (Table 3). Five 5-mL replicates of each dilution were inoculated into autoclaved 8-mL borosilicate tubes with HDPE caps. These grow-out tubes were incubated at a temperature and irradiance tested to provide rapid growth (see “Results”), and Chl*a* fluorescence was monitored with the Turner 10AU fluorometer every 2–7 days, depending on growth rate.

During the incubations, Chl*a* fluorescence in the culture tubes could increase due to an increase of cell numbers and/or due to recovery from a treatment-induced decrease in per-cell fluorescence. To account for treatment-induced changes in per-cell fluorescence that might be confounded with changes in cell number, per-cell Chl*a* fluorescence was measured with the flow cytometer before and after 5 days in the dark with or without UVC treatment. It declined strongly with increasing UVC dose in *Isochrysis*, *Phaeocystis*, and *Heterosigma* and declined below the *T*_0_ value without a dependence on UVC dose in the other taxa. The 1st percentile in a frequency distribution of per-cell fluorescence, measured immediately after the 5-day treatment regime and normalized to the *T*_0_ value, was 0.125, an 8-fold reduction. Recovery of the *T*_0_ fluorescence intensity in the light to its initial value without cell division would therefore account for less than an 8*×* increase for 99% of the population. Consequently, we set the criterion for scoring positive growth as an increase of 8*×* in *F* (but see “Results” and “Discussion”). Tubes were scored as positive for growth if fluorescence increased 8*×* above either LLD_*F*_ or the fluorescence measured immediately after inoculation, whichever was higher.

Tubes were scored as negative for growth if the criterion for a positive score was not met during the period in which a detectable increase in fluorescence would be predicted from the growth rate of cultures that did grow, with an added margin of 5 generations (see below). When all grow-out tubes had been scored as either positive or negative, the most probable number of viable cells in the undiluted sample (± the 95% confidence intervals) was determined from lookup tables (FDA 2010). The concentration of viable cells in the undiluted sample, *N*_viable_, was calculated (Eq. 5, Table 3) from MPN by normalizing to the incubation volume and reconciling the dilution range used (10^*X*^, 10^*X* − 1^, and 10^*X* − 2^) to the range of 10^−1^, 10^−2^, and 10^−3^ used in the lookup tables. Viability was expressed relative to the untreated sample as relative viability (RV, dimensionless; Table 2), the ratio *N*_viable_/*N*_0_.

## Results

### “Best practice” procedures for the SDC-MPN growth assays

We tested cell viability by monitoring growth in SDC-MPN assays using Chl*a* fluorescence. This required developing a set of best practice protocols that were constrained by rigorous and conservative quality control criteria designed to minimize experimental errors and maximize repeatability between experiments on the same species; these are summarized in Table 3.

**Fig. 1:**
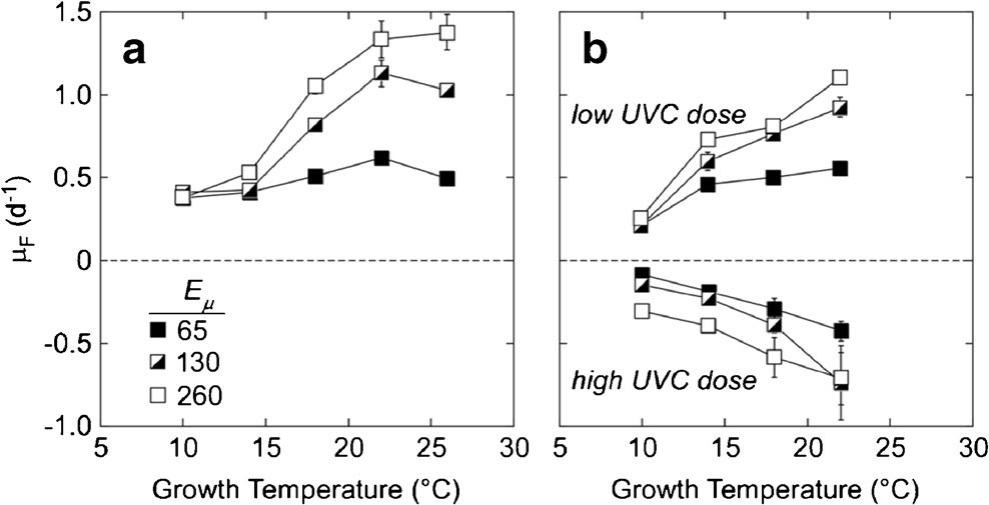
Fluorescence-based specific growth rates, *μ*_*F*_, of the diatom *Thalassiosira weissflogii* at different combinations of growth irradiance (*E*_*µ*_) and temperature following treatment. Cultures were acclimated to 130 μmol photons m^−2^ s^−1^ at 18 °C and then subjected to the experimental treatment: 5 days in darkness at 18 °C ± UVC exposure before and after the dark holding period. Data are for **a** control cells, which were not irradiated, and **b** for cells exposed to a low or high dose of UVC. Note that the growth rates for the last are negative, indicating bleaching and/or loss of cells following the return to light. Data are shown as the mean ± standard deviation of 3 replicate cultures

To optimize growth conditions in the assays while ensuring that they did not confound the outcome of tests, growth of three taxa (*Thalassiosira*, *Amphidinium*, and *Heterosigma*, Table 1) was tested in a matrix of irradiance x temperature for cultures that had been subject to a low dose of UVC (> 10^−1^ RV), a high dose (< 10^−6^ RV), or no dose (dark controls). Of the treatments tested, the highest apparent growth rates for each of the three taxa were consistently obtained at 22 °C and 260 μmol photons m^−2^ s^−1^ (Fig. 1). Critically, growth responses for each treatment were consistent over all temperatures and light intensities tested, i.e., controls and cultures subjected to the low dose of UVC always grew, and cultures subjected to the high dose of UVC always failed to grow and bleached. Consequently, the choice of growth conditions would affect only the time required to detect growth and score the test—because slower growth requires a longer assay—but would have no effect on the outcome of the test. For all other taxa tested, the growth rate at 22 °C and 260 μmol photons m^−2^ s^−1^ was tested prior to any SDC-MPN assays. The grow-outs were performed under these conditions except where *μ*_*F*_ was lower than at the conditions under which the cultures were acclimated (18 °C and 130 μmol photons m^−2^ s^−1^). This was the case for *Lingulodinium*, which was assayed under the acclimated conditions.

**Fig. 2:**
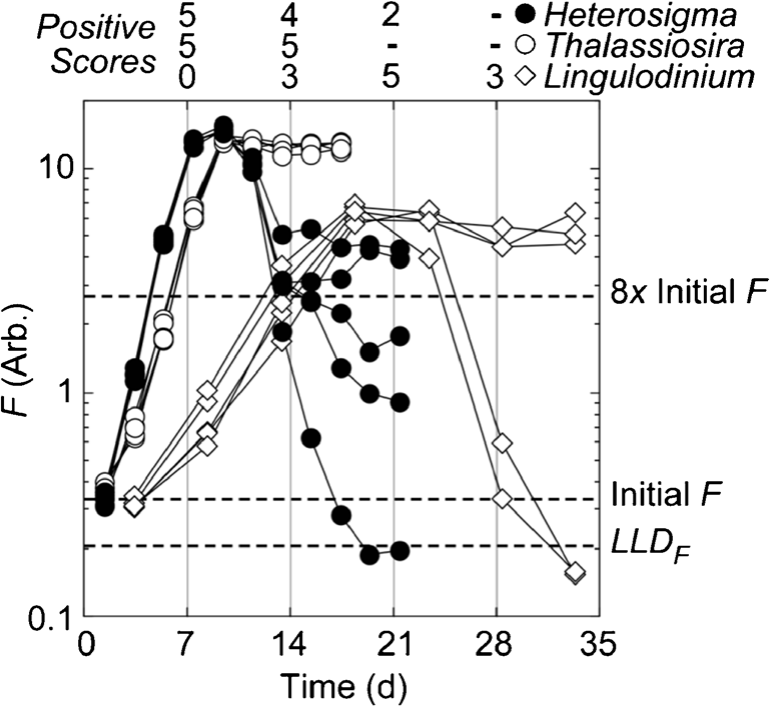
Variation in fluorescence with time during SDC-MPN assays of *Heterosigma akashiwo*, *Thalassiosira weissflogii*, and *Lingulodinium polyedra* (5 replicates per culture; only one dilution shown for each species). The number of positive scores in hypothetical single-point MPN assays terminated on days 7, 14, 21, and 28 is shown above the graph; because viable cells were present in all tubes, the correct score is 5 for each species

Patterns of growth after inoculation demonstrated that it was necessary to monitor the grow-out tubes repeatedly to obtain accurate scores, rather than measuring fluorescence on inoculation and at an arbitrarily chosen end date. This is illustrated in Fig. 2, which shows the time-courses of fluorescence in replicate tubes of two fast- and one slow-growing cultures. Of the former, growth of *Thalassiosira* would be assessed accurately as 5 positive scores if a single test were done 7 or 14 days after inoculation. However, a decline in fluorescence after 7–11 days in *Heterosigma* would result in underestimates of the true score if single tests were done at 14 or 21 days (4 and 2 positives, respectively, rather than 5). In *Lingulodinium*, the relatively slow growth and the loss of fluorescence in two of the tubes during stationary phase would lead to positive scores of 0, 3, 5, and 3 if the test were only scored once at 7, 14, 21, or 28 days after inoculation, respectively. If a scoring threshold lower than the 8*×* increase in fluorescence were chosen, some of the tabulated positive scores would increase, but the general pattern and conclusions would be the same.

**Fig. 3:**
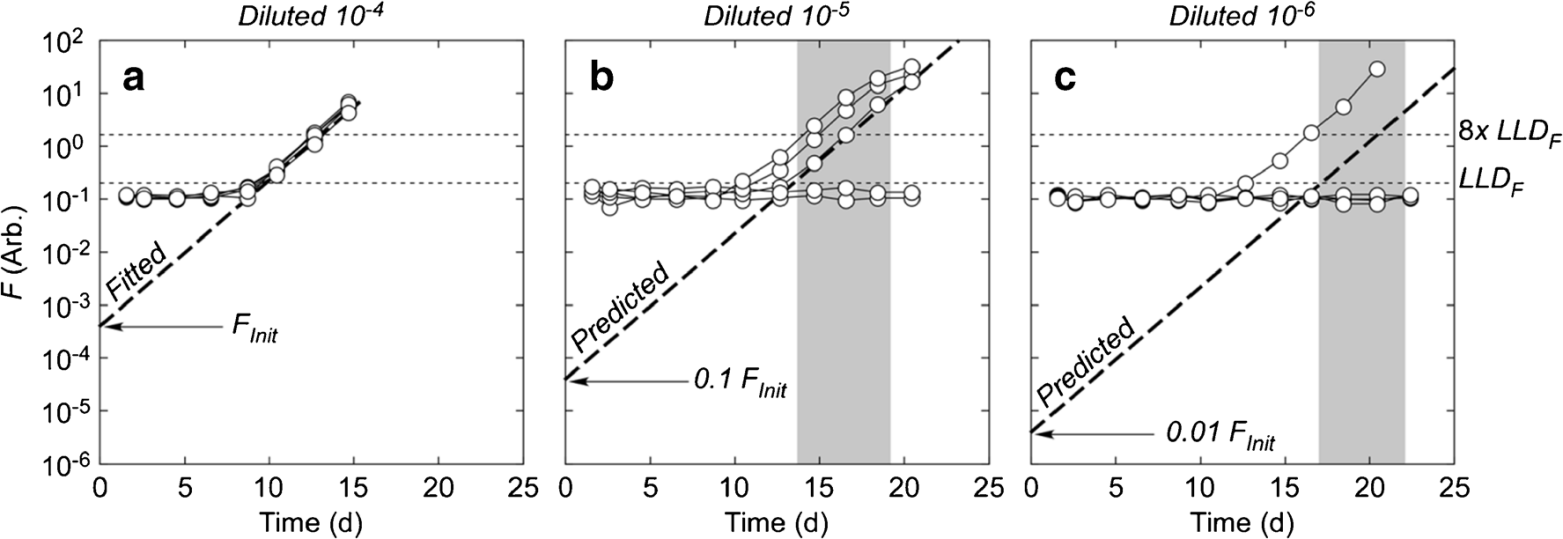
Time-courses of in vivo fluorescence in three serial dilutions of a culture of *Eutreptiella gymnastica*, with 5 replicates per dilution. Note log scales. Tubes in which fluorescence increases above 8*×* the lower limit of detection (LLD_*F*_) are scored as positive in the SDC-MPN assay. **a** The dashed line (“fitted”) is an extrapolation of the exponential growth phase, determined by regression of ln-transformed fluorescence on time for points above LLD_*F*_ (see text for details). The intercept is *F*_Init_ and the slope is *μ*_*F*_. **b**, **c** The dashed lines (“predicted”) are the expected time-courses of fluorescence for tubes that are 10 and 100 times more dilute that in **a**, respectively, assuming that a hypothetical fractional cell at the highest dilution can grow exponentially after inoculation; in practice, cultures with one or more viable cells at inoculation will show growth curves above LLD_*F*_ and tubes with no cells will not. The shaded bars indicate a 5-generation period from the time at which fluorescence is predicted to exceed LLD_*F*_. Tubes without an increase in fluorescence at the end of this period (*t*_end_, right margin of the gray bar) are scored as negatives. The final MPN score in this example is 5–3–1

It was equally important to define criteria for negative scoring to prevent a sample that had been growing slowly below the LLD from being misclassified because it was not monitored for long enough to show growth. We developed an objective method to do so, based on linear regression of the growth curve to a day-0 value. Other researchers (Redford and Myers 1951; Romero-Martínez et al. 2016) have used the intercept from the regression of a growth curve as a direct estimate of the number of viable cells—this treatment differs in using the regression as a quality-control criterion on the MPN estimate rather than as an estimate in its own right. An increase in fluorescence in the least-diluted samples can be fitted by linear regression of ln-transformed values greater than LLD_*F*_ on time (Fig. 3a). The slope of the regression is the specific growth rate, *μ*_*F*_, and the intercept, *F*_Init_, is an estimate of the fluorescence of viable cells on inoculation, assuming no lag in the growth response (see Cullen and MacIntyre 2016). Because they are all treatments of the same sample, differing only in the degree of dilution, the increase in fluorescence in any tubes containing viable cells in the second and third tier of dilutions can be predicted on two assumptions: that the growth rate is the same and that the fluorescence of viable cells on inoculation is reduced by the ratio of the relative dilutions, 10^−1^ and 10^−2^. These are the “predicted” lines in Fig. 3b, c. At sufficiently high dilution, the predicted line will correspond to the growth of a hypothetical fractional cell at inoculation, so the tubes that contain one or more cells will display growth curves above the line, consistent with the results in Fig. 3. Monitoring tubes that show no growth for the equivalent of a further 5 generations after the predicted time to reach LLD_*F*_ (*t*_end_; Eq. 6a, Table 3) provides increased certainty in concluding that there is—and will be—no growth in a tube (Fig. 3b, c). Applying the findings of the analyses summarized here, the following procedure was established: any tube in which fluorescence has exceeded 8*×* LLD_*F*_ or 8*×* the initial fluorescence by *t*_end_ is scored as positive (Eq. 7a, Table 3), and any tube that has not shown an 8*×* increase in fluorescence by this point is scored as negative (Eq. 7b, Table 3).

**Fig. 4:**
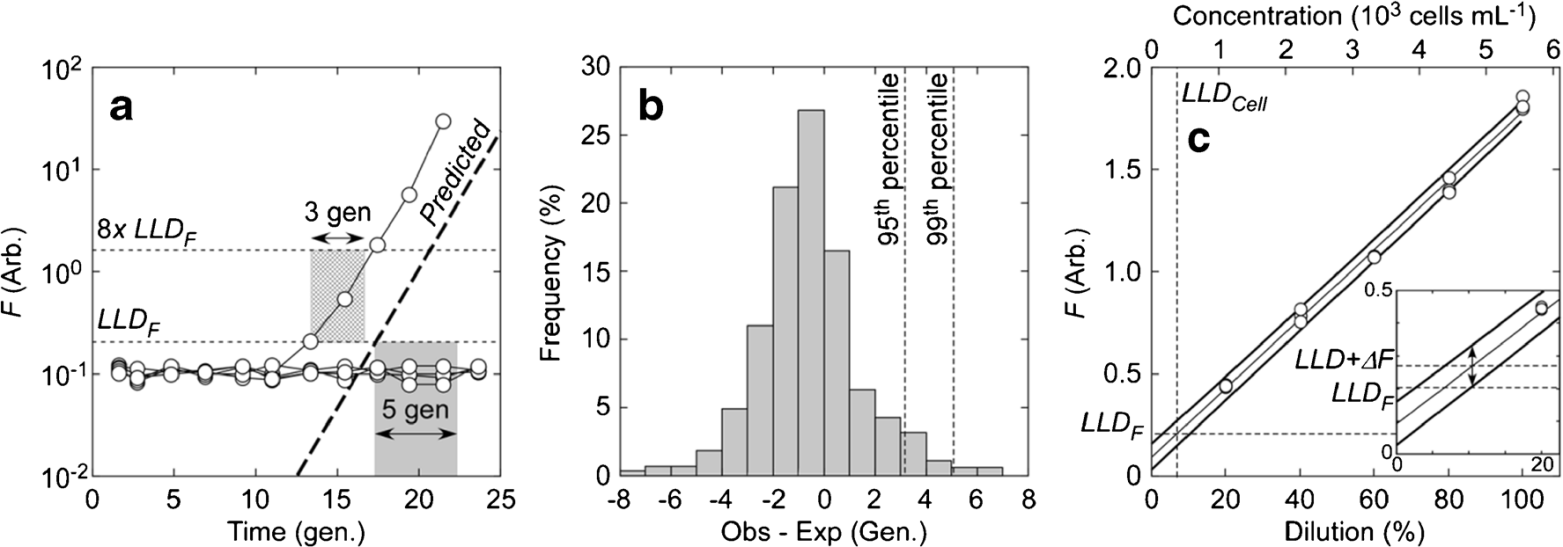
Determination of *t*_end_ in SDC-MPN assays. **a** Observed time dependence of bulk fluorescence in the most-dilute tubes in an SDC-MPN assay (10^−6^ dilution from Fig. 3). The expected growth (“predicted”; dashed line) was based on the *F*_Init_ method (Fig. 3). Time is expressed in generation equivalents, *t*_gen_ (Table 2). The duration of the 5-generation margin of error and the 3-generation (8*×*) increase in fluorescence is shown as shaded boxes. Note that observed *F* in the sole tube that exhibited growth exceeded LLD_*F*_ 3 generations ahead of the expected time. **b** Frequency distributions of observed-expected differences in the time to reach LLD_*F*_ in the second and third tiers of dilutions in all species (*n* = 910). The expected times were calculated from *F*_Init_ (Fig. 3). Both observed and expected times to LLD were calculated by regression of ln(*F*) on time and expressed in terms of the generation time in the least-diluted tubes. The 95th and 99th percentiles of the distribution (dashed lines) were 3.14 and 5.06 generations, respectively. **c** Variation in *F* with cell concentration in dilutions of a culture of *Eutreptiella gymnastica* used to estimate LLD_*F*_ (see text for details). Estimates of *F* from dilution by linear regression and the 95% confidence intervals of the estimates are shown as light and heavy lines, respectively. The minimal increase in fluorescence (Δ*F*) required to demonstrate growth is defined as the value at which the lower 95% confidence interval is equal to LLD_*F*_ (inset). In this case, LLD_*F*_ + Δ*F* was 1.35*×* LLD_*F*_ (0.43 generations). The calculation requires that there be no variation in per-cell fluorescence

The duration of *t*_end_ is determined by the 3-generation period used to define growth in the least-diluted tubes and by the 5-generation margin of observation beyond the predicted time of appearance above LLD_*F*_ in the second and third tiers of dilutions (Fig. 4a; Eq. 6). The rationale for the latter was empirical: a frequency distribution of the observed minus expected time to LLD_*F*_ in the second and third tiers of dilutions showed that 99% of tubes exhibiting growth would grow to LLD_*F*_ within 5 generations (Fig. 4b; *Y* = 5 in Eq. 6, Table 3). If the 95th percentile of the distribution were used instead, the margin would be 3 generations (Fig. 4b; *Y* = 3 in Eq. 6, Table 3). Under these conditions, *t*_end_ would be determined by the 3-generation duration of the window for any tube exhibiting growth before the predicted date (67% of the cases). For any tube exhibiting growth later than the prediction, *t*_end_ would be determined by the time required to satisfy the criterion for confirming growth.

Analysis of the data collected to define LLD_*F*_ shows that the minimum increase in *F* required to demonstrate growth is much lower than the 8*×* increase in *F* corresponding to 3 generations. The minimal increase in fluorescence required to demonstrate growth is defined as the value at which the lower 95% confidence interval (Sokal and Rohlf 2011) is equal to LLD_*F*_ (Fig. 4c). The calculated values for the different taxa (all species except *Lingulodinium*, Table 1) were 0.09–0.45 (median, 0.29) of LLD_*F*_. That is, an increase in fluorescence of less than 50% beyond that corresponding to LLD_*F*_ could be reliably detected. The period of growth required to confirm a positive score could therefore be reduced significantly below 3 generations (8*×* increase in *F*) without compromising the accuracy of the test.

### Assessment of viability following UVC treatment by SDC-MPN assay

The protocols described above were used to estimate the relative viability of 12 species at different doses of UVC. Because their sensitivity to UVC was unknown in advance, this necessitated a range-finding experiment for each to define the appropriate range of UVC doses and to determine the appropriate dilutions for the SDC-MPN assays (Eq. 4, Table 3). Based on the results of the range-finding experiment, each species was then tested over a range of UVC doses that lead to a maximum reduction in relative viability, RV, to 10^−4^–10^−6^. An SDC-MPN assay was also performed on the untreated parent culture (*T*_0_). As is expected if the SDC-MPN assay accurately enumerates actively growing cells, total measured cell concentrations in the *T*_0_ samples, measured with flow cytometry (*N*_0_), fell within the 95% confidence intervals of the estimates of viable cells in the *T*_0_ SDC-MPN assays in all cases. The limit of detectable relative viability, defined as the point at which the calculated concentration would be reduced to one viable cell in a grow-out tube, was 10^–6.3^ for *Synechococcus*, 10^–5.8^–10^−5.9^ for *Isochrysis* and *Phaeocystis*, and 10^–4.0^–10^−5.1^ for the other, larger eukaryotic taxa.

Because dose-response curves for relative viability showed both “shouldering” and “tailing” (Weavers and Wickramanayake 2001; Hijnen et al. 2006), data were fitted to a biphasic model (Ren et al. 2016) modified to allow a shoulder (i.e., the lack of effect below a threshold dose). Data were fitted to the following equations simultaneously:

8a $$ {\mathrm{RV}}_D={\mathrm{RV}}_{D=0}\kern0.5em \mathrm{for}\kern0.5em D\le {D}_{\mathrm{Th}} $$

and

8b $$ {\mathrm{RV}}_D={\mathrm{RV}}_{D=0}\cdot \left[\left(1-\alpha \right)\cdot \exp \left(-{k}_1\left[D-{D}_{\mathrm{Th}}\right]\right)+\alpha \cdot \exp \left(-{k}_2\left[D-{D}_{\mathrm{Th}}\right]\right)\right]\kern0.5em \mathrm{for}\kern0.90em D>{D}_{\mathrm{Th}} $$

where RV_*D*_ is relative viability (*N*_viable_/*N*_0_, dimensionless) for UVC dose *D* (mJ cm^−2^) applied before and after the 5-day dark hold; *D*_Th_ is a threshold dose (mJ cm^−2^) below which viability is unaffected by dose; RV_*D* = 0_ is the relative viability after treatments at or below the threshold dose—this serves as the statistical estimate for the dark-control samples that are held in darkness but without UVC exposure, as distinct from the observed value. The response to UVC doses above the threshold is biphasic and dependent on dose in exceedance of *D*_Th_. The biphasic response is partitioned by the dimensionless coefficient *α*, which varies between 0 and 1. The sensitivity coefficients *k*_1_ and *k*_2_ (mJ^−1^ cm^2^) correspond to the two inferred targets with relative influences of 1 – *α* and *α*, respectively.

In the case where *k*_1_ = *k*_2_ = *k*, the decline in viability follows first-order kinetics and Eq. 8b reduces to:

8c $$ {\mathrm{RV}}_D={\mathrm{RV}}_{D=0}\cdot \exp \left(-k\left[D-{D}_{\mathrm{Th}}\right]\right)\kern0.5em \mathrm{for}\kern0.5em D>{D}_{\mathrm{Th}} $$

Because of the nonlinear distribution of the data, these equations were fitted after log-transformation of RV_*D*_. The dose required to reduce relative viability to 10^−2^—the requirement for the IMO’s approval during land-based testing rather than the dose that would be required to meet the regulatory standard of 10 viable/living cells mL^−1^—is *D*[0.01] (mJ cm^−2^), which was obtained by solving Eq. 8 for the case where RV_*D*_ = 0.01.

Equation 8 describes a response that is a function of dose in exceedance of a threshold that is ≥0 mJ cm^−2^. Equations 8a, 8b (biphasic), or 8a, 8c (first-order) were fit simultaneously with a Levenberg-Marquardt method in Kaleidagraph 4.5 (Synergy Software) using the Boolean expression *D ≥ D*_Th_ = 1 if true, otherwise 0, with *D*_Th_ constrained to be ≥ 0. Fits for the biphasic model are reported where the RMSE for Eqs. 8a, 8b was lower than for Eqs. 8a, 8c and where the estimates of *k*_1_ and *k*_2_ were both positive. (In two cases, estimates of *k*_2_ were negative, which results in a local minimum in the response curve, with apparent increased viability at higher doses.)

**Fig. 5:**
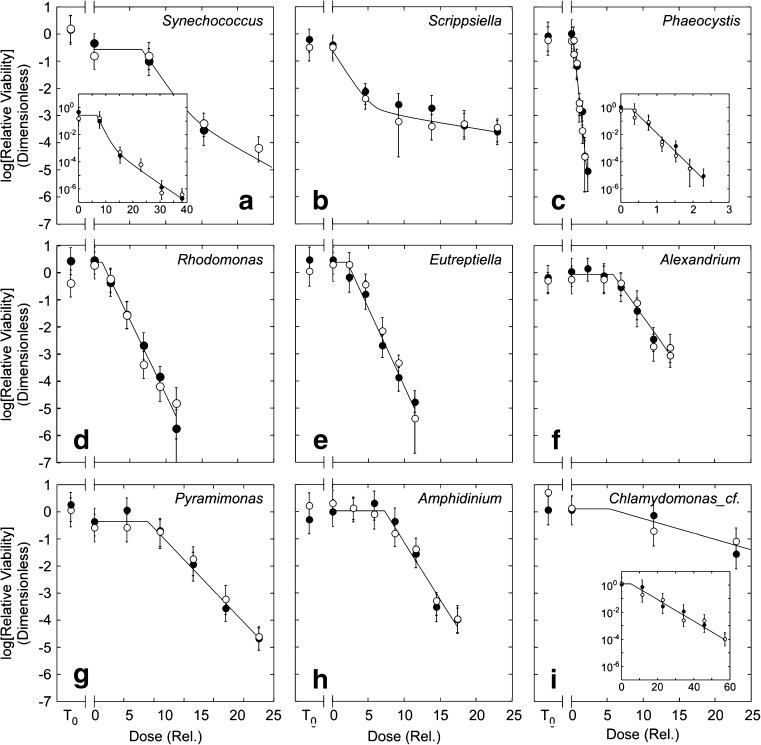
Dose-response curves of relative viability vs UVC exposure in **a** the cyanobacterium *Synechococcus elongatus*, **b** the dinoflagellate *Scrippsiella trochoidea*, **c** the haptophyte *Phaeocystis globosa*, **d** the cryptophyte *Rhodomonas salina*, **e** the euglenoid *Eutreptiella gymnastica*, **f** the dinoflagellate *Alexandrium andersoni*, **g** the chlorophyte *Pyramimonas parkeae*, **h** the dinoflagellate *Amphidinium carterae*, and **i** the chlorophyte *Chlamydomonas_*cf. sp. The UVC dose, *D*′, is given relative to the dose required to effect a reduction of 10^−2^ in relative viability in *Phaeocystis*, the most sensitive taxon. Note the different scales on inset graphs. *T*_0_ values are for cultures in balanced growth. Replicates in the response curves, shown by different symbols, are from independent cultures and were assayed up to 3 months apart from each other. Relative viability is defined as the concentration of viable cells, *N*_viable_, determined by SDC-MPN assay, normalized to the *T*_0_ cell concentration, *N*_0_, determined by flow cytometry. Error bars are the 95% confidence intervals of each MPN estimate. The lines are fits to Eq. 8. Parameter values are reported in Table 4

Coefficients of UVC sensitivity are presented as *D*′[0.01], ${D}_{\mathrm{Th}}^{\prime }$, and *k*′, after scaling to the values found for *Phaeocystis*, the taxon most sensitive to UVC (Table 4). There were widespread differences between species in the relationship between relative viability and UVC dose (Table 4, Fig. 5). These could be grouped into three categories. In the first, exemplified by *Synechococcus* (Fig. 5a) and also observed in *Thalassiosira* and *Isochrysis* (not shown), the dose-response curve was biphasic with a threshold dose (a “shoulder” as described by Hijnen et al. 2006). In the second form of response, exemplified by *Scrippsiella* (Fig. 5b) and also observed in *Heterosigma* (not shown), the response was biphasic but without a shoulder. The third form of response was a first-order (i.e., log-linear) response but only above a threshold dose, *D*_Th_. This is exemplified by *Phaeocystis* (Fig. 5c).

**Table 4:** Parameters of the dose-response curve for UVC radiation and relative viability in 9 species of phytoplankton (Fig. 5). Absolute values of the fits are proprietary, so UVC doses and sensitivity coefficients are reported relative to the dose required to effect a 10^−2^ reduction in relative viability in *Phaeocystis globosa*, the most sensitive taxon studied. Relative viability was fitted to UVC dose using Eq. 8 (see text for details). The intercept corresponds to the effects of 5 days in darkness with no UVC treatment. *D*′[0.01] is the UVC dose required to reduce relative viability by a factor of 100. Taxa are sorted by type of response (see text for details). Dose-response curves for *Isochrysis*, *Heterosigma*, and *Thalassiosira* (not reported) were all biphasic. Coefficients are reported ± the standard error of the estimate, with the exception of the intercept and threshold dose, which are reported with the 95% confidence interval. If the 95% confidence interval of the intercept or the threshold dose encompassed 0, the curve was refitted without that term. These are indicated by dashes

Response	Species	Intercept (RV_*D* = 0_, dimensionless)	Threshold dose (${D}_{\mathrm{Th}}^{\prime }$, rel.)	Sensitivity coefficient (*k*′, rel.)	Partitioning coefficient (*α*, dimensionless)	*D*′[0.01] (rel.)
Biphasic	*Scrippsiella trochoidea*	0.28 (0.13–0.61)	–	*k*_1_ 1.001 ± 0.173 *k*_2_ 0.103 ± 0.040	0.0087 ± 0.0068	3.52
	*Synechococcus elongatus*	0.26 (0.08–0.85)	6.80 (5.71–7.89)	*k*_1_ 0.924 ± 0.527 *k*_2_ 0.322 ± 0.068	0.0162 ± 0.0280	10.5
First-order	*Alexandrium andersoni*	–	5.56 (5.09–6.03)	0.871 ± 0.076		10.9
	*Amphidinium carterae*	–	7.52 (7.01–8.03)	0.913 ± 0.066		12.6
	*Chlamydomonas_cf.* sp*.*	–	5.60 (3.28–7.92)	0.173 ± 0.012		32.3
	*Eutreptiella gymnastica*	–	3.06 (2.51–3.61)	1.46 ± 0.101		6.55
	*Phaeocystis globosa*	–	0.22 (0.15–0.29)	5.90 ± 0.334		1.00
	*Pyramimonas parkeae*	0.45 (0.27–0.73)	8.15 (7.55–8.75)	0.669 ± 0.034		13.8
	*Rhodomonas salina*	–	0.96 (0.41–1.51)	1.25 ± 0.070		5.32

The sensitivity of an organism to UVC treatment, e.g., as represented by *D*′[0.01], the dose required to reduce RV by a factor of 100, scaled to the minimum value found among the taxa (Table 4), varied by 32*×* (Table 4). This measure is determined by the threshold (${D}_{\mathrm{Th}}^{\prime }$), the sensitivity to UVC doses above the threshold (*k*′), and effects of the 5-day dark treatment (RV_*D* = 0_). With the exception of *Heterosigma* (not shown) and *Scrippsiella*, all taxa had scaled threshold doses, ${D}_{\mathrm{Th}}^{\prime }$, that were significantly higher than 0 (Fig. 5; Table 4), varying by 37*×* in magnitude (Fig. 5, Table 4). The lowest value was observed in *Phaeocystis* and the highest value was observed in *Pyramimonas*. The sensitivity coefficients, *k*′, which are also scaled by the dose required to effect a 100*×* reduction in viability in *Phaeocystis*, varied by more than an order of magnitude, too. The range was 34*×* (Table 4) for the first-order fits. These were not significantly correlated with the threshold dose (*p* = 0.10; *n* = 7 for species with first-order responses—data are not included for species with biphasic responses because of differences in the model structures). Changes associated with 5 days in the dark without UVC treatment were indicated by significant non-0 intercepts for 5 species (3 shown in Table 4): RV_*D* = 0_ was < 1 in *Pyramimonas*, *Scrippsiella*, and *Synechococcus* (Table 4) and also in *Heterosigma* and *Isochrysis* (not shown). None of these indices of UVC sensitivity was significantly (*p* < 0.05) correlated with cell size, as ACD, in the eukaryotic taxa—ACD could not be measured accurately in *Synechococcus*, which is, nonetheless, much smaller than the eukaryotes. The *D*′[0.01] in *Synechococcus* was in the mid-range of values among species.

The wide differences in both the form of response and sensitivity to UVC were repeatable for independent tests of the same species. Replicate data for each taxon (different symbols in Fig. 5) were collected on independent cultures at intervals of weeks to months. As the cultures were all in balanced growth—i.e., fully acclimated to the growth conditions—there should have been no consistent differences between the replicate curves. This was confirmed by comparison of the normalized residual errors in Eq. 8 at each dose between different test dates: in all cases, there was no significant difference (ANOVA, *p* > 0.05).

We were unable to measure a dose-response curve for *Lingulodinium* (Table 1). The cultures were handled with extreme care while mixing and pipetting, and the number of viable cells in the *T*_0_ sample was in good agreement with the measured cell concentration (*N*_viable_ = 3400 cells mL^−1^, 95% CI 1160–8000 cells mL^−1^, vs *N*_0_ = 3543 cells mL^−1^). Even so, growth was detected in only 4 of the 12 samples subjected to the experimental treatments. Relative viability in these was low (2.3 × 10^−4^–2.0 × 10^−3^) and was not significantly correlated with UVC dose (*p* = 0.51). Although viable cells were detected in some of the UV-treated samples, no viable cells were detected in the dark controls.

### Delayed repair of UVC damage

The descriptions of dose-response above are predicated on the assumption that there was no delay in repair and recovery beyond the duration of the incubations—i.e., that the observed estimates of viability are an accurate reflection of the rates of disinfection. Delayed repair could be expressed as incomplete recovery of UVC-impaired growth rate during the incubation, thereby compromising the determination of *t*_end_ (Eq. 6, Fig. 3), or a delay in the commencement of recovery—i.e., a lag, sensu Cullen and MacIntyre (2016)—long enough to prevent accurate detection of growth by the end of the assay.

**Fig. 6:**
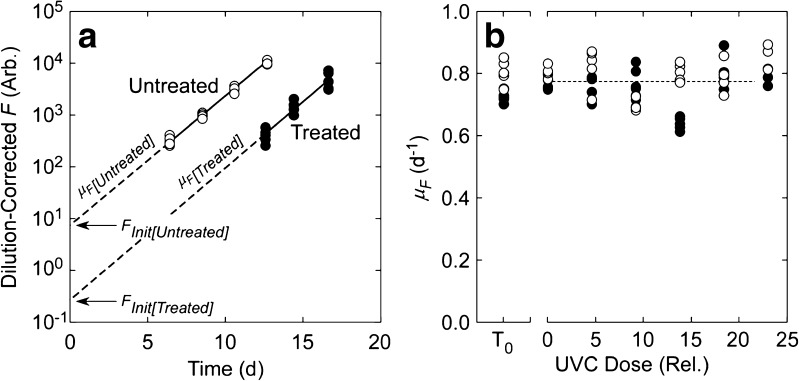
**a** Variation in fluorescence, *F*, in the upper tier of SDC-MPN assays of untreated (*T*_0_) and UVC-treated samples of *Eutreptiella gymnastica* (5 replicates for both assays). Only values above the limit of detection, LLD_*F*_ (see Fig. 3), are shown. Regression of ln-transformed data gives the specific growth rate (*μ*_*F*_, day^−1^) and intercept (*F*_Init_, dimensionless). **b** Variation in specific growth rates with normalized UVC dose, *D*′ (cf. Fig. 5), in *Pyramimonas parkeae*. Data are from positively scoring tubes from the least-dilute tier of MPN assays. Data for the corresponding (untreated) *T*_0_ samples are shown for comparison. Open and closed symbols are for independent experiments

Incomplete recovery of growth rate is addressed through an analysis of the growth curves in the SDC-MPN dilutions. The specific growth rate (*μ*_*F*_, Fig. 6a) reflects integrated cellular function and persistent debilitation (i.e., incomplete recovery) would result in significantly reduced growth rates as a function of UVC dose. In all species except *Scrippsiella*, there was no significant (*p* < 0.05) dose dependence of growth rates in the SDC-MPN assays (e.g., Fig. 6b). Even in *Scrippsiella*, there was only a significant decline in growth rate at the 2 highest doses, where relative viability was reduced below 0.075% (Fig. 5). The lack of dose dependency indicates that when growth became observable, i.e., when fluorescence rose above LLD_*F*_ (see Fig. 3), the surviving cells had recovered fully from any damage imposed by the treatment.

**Fig. 7:**
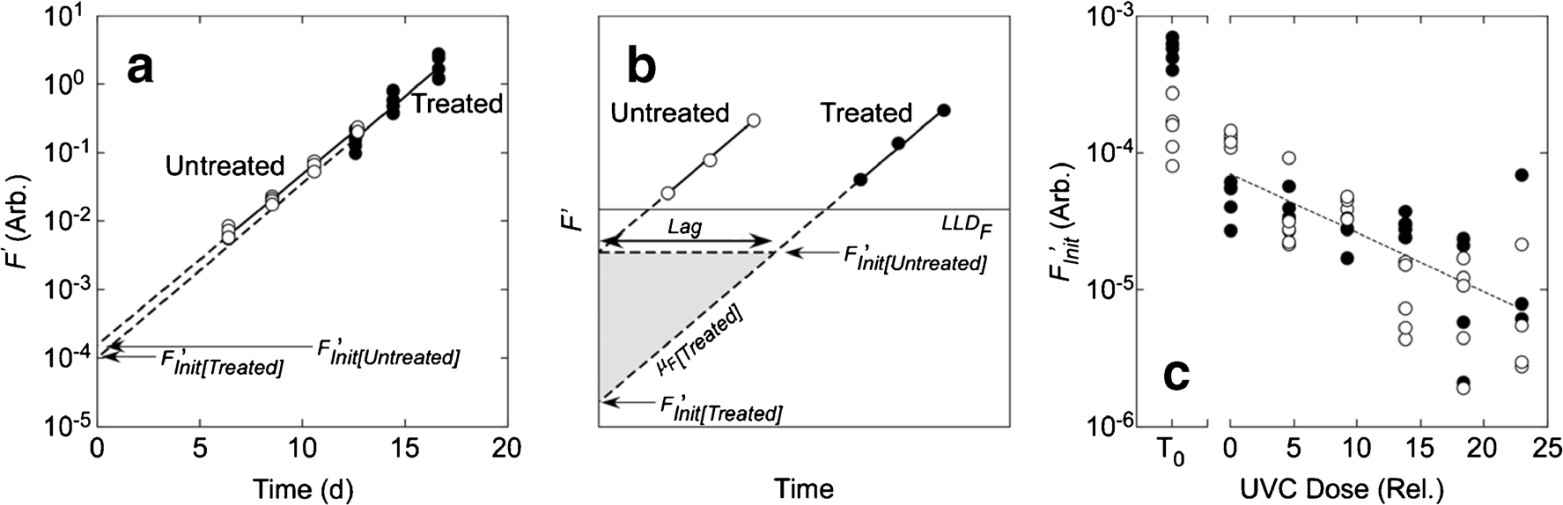
**a** Variation in dilution-corrected fluorescence normalized to the estimated number of viable cells, *F′*, in the upper tier of MPN growth assays of untreated (*T*_0_) and UVC-treated samples of *Eutreptiella gymnastica* (same data as Fig. 5a; 5 replicates for each treatment). Only values above the limit of detection, LLD_*F*_ (see Fig. 3), are shown. Regression of ln-transformed data gives the intercept (${F}_{\mathrm{Init}}^{\prime }$, dimensionless). Close agreement in the two regressions is consistent with no lag in the resumption of growth of viable cells after treatment. The subscripts [Treated] and [Untreated] discriminate between estimates from these two treatments. **b** Variation in *F′* with time illustrating the lag phase in recovery from a hypothetical experimental treatment. Regression of ln-transformed data gives the specific growth rate (*μ*_*F*_, day^−1^) and intercept (${F}_{\mathrm{Init}}^{\prime }$, dimensionless). The lag is the period (double-headed arrow) required for recovery of *F′* in the treated sample to the *T*_0_ value for the untreated culture. This is calculated from the difference between the two values of ${F}_{\mathrm{Init}}^{\prime }$ and *μ*_*F*_ for the treated sample: in practice, it encompasses any trajectory through the stippled area between the two values. **c** Variation in ${F}_{\mathrm{Init}}^{\prime }$ with UVC dose in *Pyramimonas parkeae*. Data are from positively scoring tubes from the least-dilute tier of MPN assays. Data for the corresponding (untreated) *T*_0_ samples are shown for comparison. UVC dose is given in relative units (cf. Fig. 5). Open and closed symbols are for independent samples

The stability of observed growth rates as a function of UVC dose indicates that if for any species there was a period of repair and recovery before growth resumed, i.e., a lag, it must have occurred before growth was detectable. The upper limit of the recovery period is therefore the time taken for fluorescence to reach LLD_*F*_ in the least-diluted tubes of each SDC-MPN assay for a given UVC dose. The duration of the lag can be estimated by comparison of *F*_Init_ in treated samples with *F*_Init_ in the corresponding untreated (*T*_0_) samples, with both normalized to the concentration of viable cells in the sample: *F* ^′^ = *F*/*N*_viable_ and ${F}_{\mathrm{Init}}^{\prime }$ = *F*_Init_/*N*_viable_ (Fig. 7a). In untreated cultures, the cells are demonstrably all viable (MacIntyre and Cullen 2016), so ${F}_{\mathrm{Init}}^{\prime }$ should accurately reflect the contribution of viable cells at time 0. If there is a dose-dependent delay in growth, effectively shifting the growth curve to the right, the value of ${F}_{\mathrm{Init}}^{\prime }$ in a treated culture will decline below ${F}_{\mathrm{Init}}^{\prime }$ in the untreated culture. This is illustrated in Fig. 7b. In 8 of the 12 species tested, there was a significant (*p* < 0.05) decline with UVC dose in log-transformed ${F}_{\mathrm{Init}}^{\prime }$ in treated cells (e.g., Fig. 7c), consistent with a progressive lag before recovery in growth rate. The exceptions were *Alexandrium*, *Isochrysis*, *Synechococcus*, and *Thalassiosira*.

**Fig. 8:**
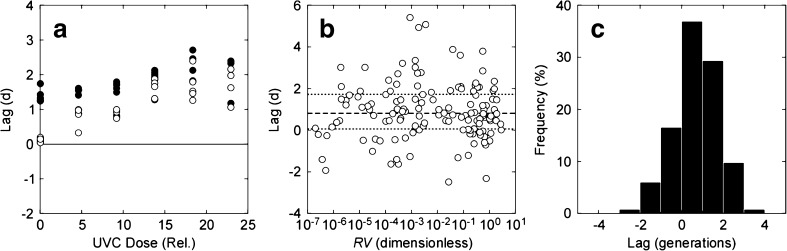
**a** Variation in calculated lag periods in positively scored tubes from the least-dilute tier of SDC-MPN assays of *Pyramimonas parkeae* as a function of UVC exposure (same data as in Figs. 6b and 7c ). Data are from positively scoring tubes from the least-dilute tier of the assays. Data for the corresponding (untreated) *T*_0_ samples are shown for comparison. UVC dose is given in relative units (cf. Fig. 4). Open and closed symbols are for independent samples. Lag was correlated (*R* = 0.65, *p* < 0.05) with UVC dose. **b** Variation in calculated lag periods (mean of 1–5 tubes per experimental exposure) with relative viability, RV, in the 12 species for which dose-response curves were measured (*n* = 133). There was no correlation (*p* = 0.52) between the variables. The dashed line is the median of the estimated lags and the dotted lines are the first and third quartiles of the data distribution. **c** Frequency distribution of estimated lag periods, expressed in generation-time equivalents (see text), for the same data

If repair and subsequent growth are assumed to be sequential, the duration of a lag period in which repair occurs can be calculated as the time that it takes for ${F}_{\mathrm{Init}}^{\prime }$ in treated cells to increase to the value in untreated (*T*_0_) cultures (Fig. 7b). Assuming exponential growth at the same rate observed when fluorescence is above LLD_*F*_:

9 $$ \mathrm{Lag}=\ln \left(\frac{F_{\mathrm{Init}}^{\prime}\left[\mathrm{Untreated}\right]}{F_{\mathrm{Init}}^{\prime}\left[\mathrm{Treated}\right]}\right)\cdot {\mu}_F{\left[\mathrm{Treated}\right]}^{-1} $$

where Lag is the duration of the lag phase (days); ${F}_{\mathrm{Init}}^{\prime }$[Treated] and ${F}_{\mathrm{Init}}^{\prime }$[Untreated] are the mean value of ${F}_{\mathrm{Init}}^{\prime }$ from the upper tier of dilutions of untreated samples and the value from a single tube of a treated sample, respectively; and *μ*_*F*_[Treated] is the growth rate in the treated sample. Estimates of lag time are negative if the value of ${F}_{\mathrm{Init}}^{\prime }$ in a treated sample is higher than the mean value in the untreated samples. The relationship between lag phase and UVC dose for *Pyramimonas* (Figs. 6b and 7c) is shown in Fig. 8a. Values ranged from −0.1 to 2.0 days. For comparison, it took 4–12 days for the same tubes to reach LLD_*F*_. Only 2 species, *Pyramimonas* and *Eutreptiella*, had lag phases that were significantly (*p* < 0.05) correlated with UVC dose or with log-transformed relative viability. Even so, the variability—as the range of estimates from a second-order polynomial fit to the data—was low compared to the time required for the cultures to reach LLD_*F*_: 1.2 vs 7.2 days in *Pyramimonas* and 2.9 vs 10.6 days in *Eutreptiella*.

There was no significant correlation between the duration of the lag phase and log-transformed relative viability for all species combined (Fig. 8b). Note that in 22% of the cases, the estimated lag time is negative, not unexpected for a calculation that is sensitive to uncertainty in the estimate of *μ*_*F*_ and where the estimates of *μ*_*F*_ in the treated and untreated cultures were very close (cf. Fig. 6a, b). The highest estimate of the lag time was 5.4 days but 58% of the estimates were < 1 day and 81% were < 2 days. For comparison, the time required to reach LLD_*F*_ in the same samples was 3–26 days (median 9 and mean 10 days). When the lag time was expressed in generation-time equivalents by dividing by ln(2) ⋅ *μ* ^−1^, the median value was 0.7 generations (Fig. 8c). The range was − 2.0 to 3.0 generations (mean 0.8): 59% of the values were < 1 generation, and 88% of the values were < 2 generations.

## Discussion

Our application of the SDC-MPN method to assess loss of viability in a wide diversity of taxa (12 species from 7 divisions) confirms that UVC is an effective biocide for phytoplankton within the range of doses applied in municipal water treatment (USEPA 2006; NWRI 2012). Quantitative parameterization of the dose-response curves showed that there were wide differences in the pattern and magnitude of response to UVC (Fig. 5, Table 4). The finding depends critically on the development of defensible “best practice” criteria for the SDC-MPN assay.

### Best practice criteria for SDC-MPN assays

By definition, the SDC-MPN assay is a quantitative test of the ability of cells to proliferate by cell division. As such, it is the appropriate assay for a stressor such as UVC, for which the primary mode of action is damage to nucleotides (Sinha and Hader 2002) and the subsequent reduction in growth potential (Breeuwer and Abee 2000). Because the assay is based on the ability to reproduce, rather than a proxy of a vital function, it is equally appropriate for assessing the effect of any treatment that is intended to render microorganisms harmless. The SDC-MPN method has been used to estimate the loss of viability due to UVC treatment (Oemcke et al. 2004; First and Drake 2014; Chaudhari et al. 2015; Wright and Welschmeyer 2015; Casas-Monroy et al. 2016), among other applications. However, to our knowledge, none of these studies has developed a formal best practice protocol that includes, critically, the definition of an appropriate duration for testing: reported tests were terminated at an arbitrary end point that ranged from 7 days (determination by variable fluorescence, First and Drake 2014) to “1 to 2 months in summer and 2 to 4 months in winter” (determination by microscopy, Knight-Jones 1951). Only by defining an appropriate end point for the assay can the absence of growth be ascribed with confidence to the absence of viable cells in a tube rather than an inability to detect slowly growing cells. We have described the formalism of defining the duration of the test in Table 3 (Eqs. 3–7) after verifying that the growth conditions were optimal for each culture that was tested.

The criteria for defining positive and negative scores (Eq. 7, Table 3) are objectively derived. While these are specific to detection by fluorescence, the same principles can be applied to other means of detecting growth. By definition, growth cannot be detected if it is invisible to the detector being used (i.e., is below the LLD), which sets the lower limit of a change in sensor output that can be used to demonstrate positive growth. The criterion that we adopted for detection by fluorescence—an increase of 8*×* above LLD_*F*_ or the initial fluorescence, whichever was higher—is an aggressively stringent criterion. It was based on changes in per-cell fluorescence that might bias interpretation of fluorescence as cell number *if they occurred when fluorescence readings were higher than LLD*_*F*_. Per-cell fluorescence (*F* cell^−1^) is likely to vary systematically throughout growth in a batch culture with up- and downregulation of the absorption cross section and quantum yield of fluorescence in response to changes in light and nutrient availability (Falkowski et al. 1981; Kolber et al. 1988; Geider et al. 1993). In practice, though, changes during the period of exponential growth are likely to be minimal compared to recovery from stresses imposed by the prolonged darkness and/or UVC. The time required to affirm positive scores could be reduced, with the attendant savings in personnel time and effort, by accepting a less demanding threshold, and there are good grounds for doing so.

As the cultures all had consistent growth rates, based on measurements of *F* above the LLD—with the exception of samples of *Scrippsiella* subjected to the two highest UVC doses—it is logical to assume that any repair and recovery from the imposed stresses was complete before growth was detectable. The estimated period for repair and recovery was 1–2 days in the vast majority (81%) of samples, consistent with the rapid rate of repair of cyclobutane pyrimidine dimers induced by UVC treatment in the chlorophyte *T. suecica* (Hull et al. 2017), and significantly shorter than the average of 10 days required to reach the LLD. It is reasonable to assume that under these conditions, *F* cell^−1^ is constant and so increases in cell concentration could be assessed based on the observations of *F*. A pragmatic alternative to the 8*×*-LLD_*F*_ criterion would be to accept an increase in *F* of 50% (based on the 95% confidence intervals of mean *F* cell^−1^ in independent cultures of 0.07–0.20*×* the mean) above LLD_*F*_ as evidence of a positive score. Adoption of the more relaxed criterion would reduce monitoring time of tubes that score positive by several days and would eliminate redundant monitoring after growth was reliably detected.

### Optimizing the precision of SDC-MPN assays

The underlying principle of the SDC-MPN assay is that the concentration of viable cells is diluted to the point at which a statistically interpretable number of tubes should contain no viable cells. This condition is satisfied when the dilution series encompasses a concentration of 1 viable cell per tube (Cochran 1950), a condition that can be satisfied if the likely range of *N*_viable_ can be constrained with confidence. Because the concentration of viable cells is generally not known, the range of the dilution series—(DR)^*q* − 1^, where DR is the dimensionless dilution ratio (fixed at 10 in this study; Eq. 4, Table 3) and *q* is the number of tiers—should be large enough to ensure that no experiment yields all sterile or all fertile tubes. To accommodate uncertainty in the estimate of *N*_viable_, the range can be expanded by increasing the number of tiers, at the cost of requiring additional resources. The dilution ratio can be increased, but at the expense of the method’s precision, which can be improved with compensatory increases in the number of replicate tubes per tier (Cochran 1950). Given that increasing the number of dilutions, tiers or tubes per tier can require a significant investment of time and resources, it is useful to examine the quantitative relationships between the precision of SDC-MPN and the design of the test.

Hurley and Roscoe (1983) presented a general equation to explore the influences of experimental design on the precision of the estimate of *N*_viable_:

10 $$ {\sigma}_{\ln \left({N}_{\mathrm{viable}}\right)}={\left[{\left({N}_{\mathrm{viable}}\right)}^2\cdot {\sum}_{i=1}^q\frac{{\left[V\cdot \mathrm{CF}(i)\right]}^2\cdot r}{\exp \left[V\cdot \mathrm{CF}(i)\cdot {N}_{\mathrm{viable}}\right]-1}\right]}^{\raisebox{1ex}{$-1$}\!\left/ \!\raisebox{-1ex}{$2$}\right.} $$

where ${\sigma}_{\ln \left({N}_{\mathrm{Viable}}\right)}$ is the standard error of ln(*N*_viable_), *V* (mL) is the volume incubated (Table 2), *q* is the number of tiers with *r* replicates per tier, and CF(*i*) (dimensionless) is the concentration factor in tier *i*. Each value of CF(*i*) is the concentration factor in tier *i*, determined from CF in the least-diluted tier and the dilution ratio between tiers, DR (dimensionless). In this study, the former is 10^*X*^ and the latter is 10 (Eq. 4, Table 3). Different dilution ratios can be accommodated in Eq. 4 by using different bases for the power function that defines DR.

A sensitivity analysis was used to examine the effect of varying *q* and *r* on the expected precision of the estimates of *N*_viable_. It included the less important influences of *V* and *N*_viable_. The analysis used 70,400 combinations of input variables, including those used by testing organizations charged with type approval of BWMS (e.g., IMO 2016, see Table 5). The dilution ratio and number of tubes explained 96% of the variability in a log-transformed regression (70,397 *df*). Expressing the independent variables in linear form:

11 $$ {\sigma}_{\ln \left({N}_{\mathrm{viable}}\right)}=0.65\cdot \frac{{\mathrm{DR}}^{0.266}}{\sqrt{r}} $$

**Table 5:** Input variables for an analysis of the influence of experimental configuration on the precision of SDC-MPN (Fig. 9). A range of nominal concentrations of viable cells, *N*_viable_, was used in Eq. 10, along with all combinations of the input variables listed below. Examples of configurations used by three testing organizations (DHI, Denmark and Singapore; NIVA, Norway; and Golden Bear, USA) come from submissions to IMO (IMO 2016). The concentration factor for the least-dilute first tier, CF(1), was calculated from *N*_viable_ and the dilution ratio DR: the original sample is diluted by DR successively until the number of detectable organisms per tube, *V* ⋅ CF(1) ⋅ *N*_viable_, is ≤DR. Consequently, the number of viable organisms per tube in tier 1 is ≥ 1.0 and in tier 2 it is ≤ 1.0

Input variable	Input values (total number)	Nominal configurations		
		DHI	NIVA	Golden Bear
Concentration (*N*_viable_, cells mL^−1^)	1–20, step = 1 1000–20,000, step 1000 (40)			
Culture volume (*V*, mL)	1–10, step = 1 (10)	6	6	3
Number of tiers (*q*)	3–8, step = 1 (6^a^)	3	3–5	7
Replicate tubes per tier (*r*)	3–10, step = 1 (8)	3 replicates 5 each	3–5	5
Dilution ratio (DR)	2 to 10, step = 1	10	10	6
Initial dilution (CF(1))	Calculated			

^a^Number of tiers is restricted to a total dilution range of 100 ≤ DR^*q* − 1^ ≤ 10,000

The result is generally consistent with the simpler relationship presented by Cochran (1950), with a mean ratio (Eq. 11/Cochran) of 0.93, s.d. = 0.03. The lower and upper 95% confidence limits on an MPN estimate of *N*_viable_ are ${N}_{\mathrm{viable}}/\exp \left(2{\sigma}_{\ln \left({N}_{\mathrm{viable}}\right)}\right)$ and ${N}_{\mathrm{viable}}\cdot \exp \left(2{\sigma}_{\ln \left({N}_{\mathrm{viable}}\right)}\right)$, respectively. The factor $\exp \left(2{\sigma}_{\ln \left({N}_{\mathrm{viable}}\right)}\right)$ serves as an appropriate substitute for a standard error (cf. Cochran 1950). For the design used in this study, DR = 10 and *r* = 5; the factor for estimated 95% confidence intervals is 2.9, generally consistent with the tabulated SDC-MPN confidence limits for individual experiments (Fig. 5).

**Fig. 9:**
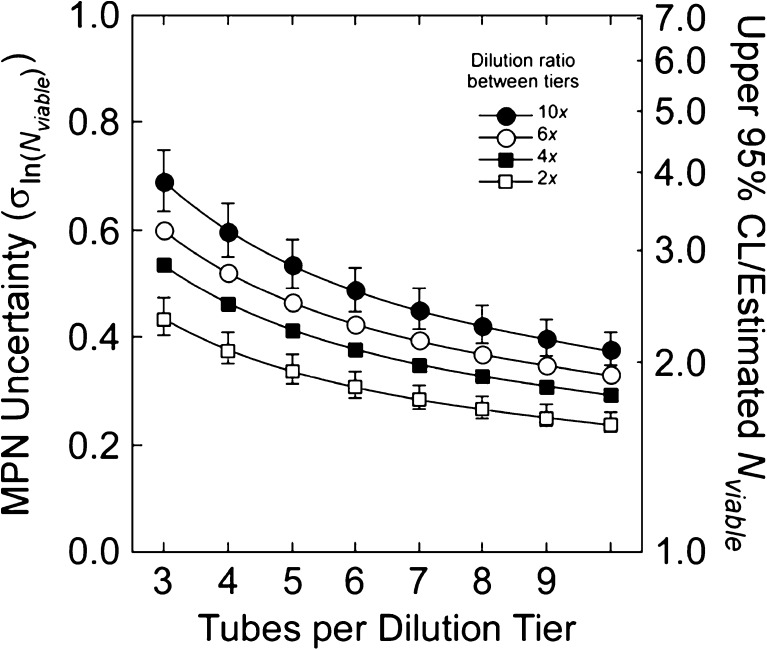
Relative uncertainty in estimates of *N*_viable_ from SDC-MPN, ${\sigma}_{\ln \left({N}_{\mathrm{viable}}\right)}$, as a function of the dilution ratio (DR) and number of tubes per dilution tier (*r*), calculated according to Eq. 10 (Hurley and Roscoe 1983). The second *y*-axis shows the ratio of the upper 95% confidence limit to the estimate of *N*_viable_, $\exp \left(2{\sigma}_{\ln \left({N}_{\mathrm{viable}}\right)}\right).$ Most of the variability in 70,400 solutions was explained by DR and *r*, shown here. The median value for ${\sigma}_{\ln \left({N}_{\mathrm{viable}}\right)}$ is plotted for each combination; vertical bars show 5th and 95th percentiles for DR = 10 (1200 solutions) and 2 (400 solutions). The ranges for DR = 4 and 6 are not shown; they are intermediate. A general solution is presented in Eq. 11

Precision is maximized by increasing the number of replicate tubes (*r*) and minimizing the dilution ratio (DR), as illustrated in Fig. 9. Doubling the number of tubes from 5 (this study) to 10 and reducing the dilution ratio from 10 (this study) to 2 would reduce the mean uncertainty in the estimate of viable cells (i.e., ${\sigma}_{\ln \left({N}_{\mathrm{Viable}}\right)}$) by 56% (Fig. 9). This would reduce the ratio of the 95% confidence interval from 0.26–3.86*×* the estimate to 0.63–1.59*×* the estimate (medians in Fig. 9). A reduction in DR would necessitate increasing the number of tiers in the assay (*q*) if *N*_viable_ were not well constrained. Consequently, the improvement in precision would come at the expense of personnel time in monitoring the increased number of tubes, *q* × *r*.

### Minimizing the duration of SDC-MPN assays

For logistical reasons, it is advantageous to minimize *t*_end_. With the current criteria, *t*_end_ is determined by the 5-generation safety margin after the most dilute sample is predicted to reach the LLD rather than the 3-generation (8*×* increase) period taken to verify growth when fluorescence is higher than the LLD (Eqs. 6 and 7, Table 3). Only reducing the duration of the safety margin would reduce the duration of the test, *t*_end_. This is predicated on three factors, the specific growth rate, the number of viable cells in the sample, and the sensitivity of the sensor used to monitor growth (Eq. 6, Table 3, see also Cullen and MacIntyre 2016). The duration of tests in this study was constrained by the availability of a sensitive instrument and ranged from 15 to 90 days where *N*_viable_ was 10 cells mL^−1^, and considerably longer (19–115 days) where the predicted concentration of viable cells was reduced to the limit of the test for 5-mL volumes, 0.36 cells mL^−1^. Reducing the length of the safety margin from 5 to 3 generations (corresponding to 99 to 95% confidence in detecting late-growing tubes in the most-diluted tier of the assay; Fig. 4b), enhancing growth rate (Fig. 1), and using a more sensitive detector (i.e., with a lower LLD_Cell_) would all shorten the duration of the test, with the attendant savings in effort and time. We consider these in turn.

**Fig. 10:**
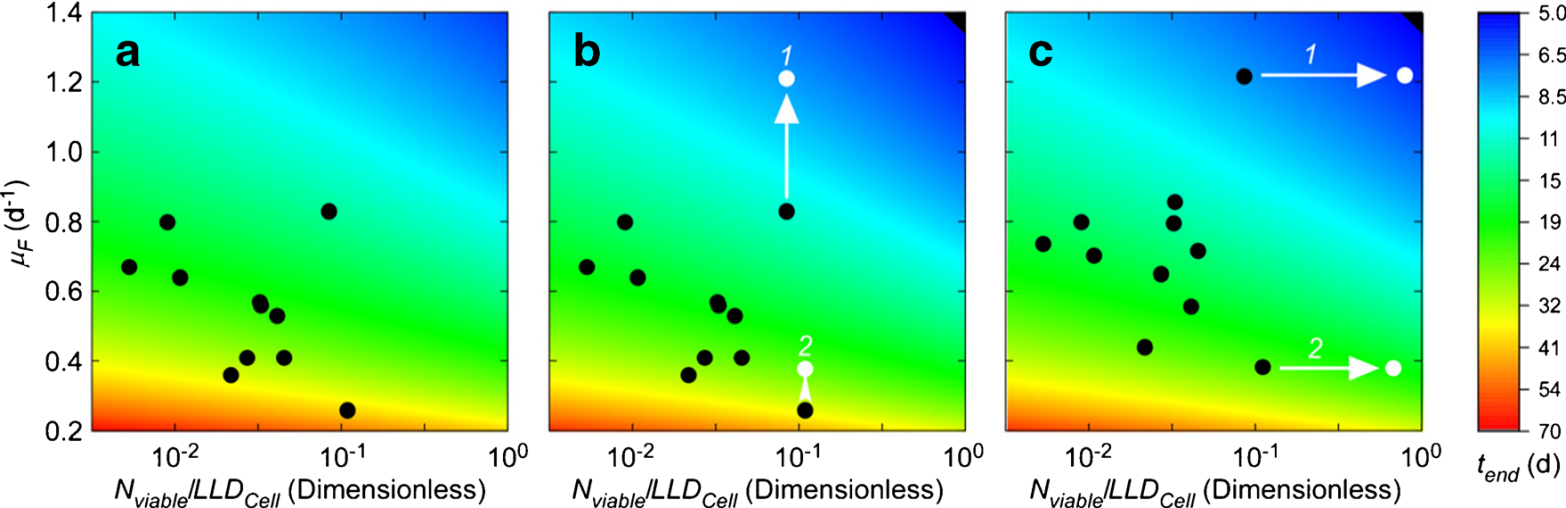
Variation in *t*_end_, the time required to complete a 3-tiered assay, as a function of growth rate (*μ*_*F*_) and the starting concentration of viable cells (Eq. 6, Table 3). The latter is expressed relative to the lower limit of detection for the strain (*N*_viable_/LLD_Cell_). Note the log scale for time. The duration of the assay is shortest under conditions of high initial concentration of viable cells (*N*_viable_) and/or high sensitivity of detection (low LLD_Cell_) and high growth rate. The points overlaid on the plot are the values for the eukaryotic taxa in Table 1, except *Lingulodinium*, with a starting concentration of 10 cells mL^−1^ and the measured LLD_Cell_. An increase in growth rate and/or a higher sensitivity results in shorter assay duration. This is illustrated for the fastest- and slowest-growing taxa, *Thalassiosira weissflogii* and *Alexandrium andersoni*. **a** Values of *t*_end_ are based on the growth rate in balanced growth (Table 1), LLD_Cell_ determined for a Turner 10AU fluorometer, and a 5-generation margin for detection (Eq. 6b). **b** Estimates as in **a** but for a 3-generation margin for detection. The white arrows and points show the reductions in *t*_end_ achieved for *Thalassiosira* (1) and *Alexandrium* (2) by optimizing the irradiance and temperature conditions in the assay to maximize growth rates. **c** Estimates as in **a** but using optimized growth rates (Fig. 1). The white arrows and points show the reductions in *t*_end_ for *Thalassiosira* (1) and *Alexandrium* (2) achieved by using a customized Satlantic FIRe fluorometer with limits of detection c. one order of magnitude below those of the 10AU

We calculate values of *t*_end_ for the third tier of an MPN assay for all eukaryotic species on which dose-response curves were measured (i.e., all except *Lingulodinium*, Table 1) using Eq. 6b. The starting concentration of viable cells, *N*_0_, was set at 10 cells mL^−1^; *μ*_*F*_ was the acclimated growth rate; and LLD_Cell_ was measured (see Fig. 4c). The calculation uses a 5-generation safety margin (Table 3). The calculated values of *t*_end_ range from 13 to 40 days (median 21 days), as shown in Fig. 10a. Reduction of the safety margin to 3 generations reduces the corresponding values of *t*_end_ to 11–34 (median 18) days (Fig. 10b). The reduction is disproportionately weighted to slow-growing cells because it scales with *μ*_*F*_^−1^ (Table 3, Eq. 6).

Because *t*_end_ is inversely proportional to growth rate, the duration can be shortened by increasing the growth rate. Doing so by optimizing the growth conditions of temperature and irradiance (cf. Fig. 1) reduces *t*_end_ by a factor proportional to [change in *μ*_*F*_]^−1^ (Eq. 6, Table 3). This is illustrated in Fig. 10b for *Thalassiosira* and *Alexandrium*, the fastest- and slowest-growing cultures used in this analysis, respectively. Optimization of growth rate reduced *t*_end_ from 11 to 7 days in the former and from 34 to 23 days in the latter, both calculated with a 3-generation safety margin. Any attempt to increase growth rate by increasing temperature and/or irradiance should be tested before conducting the assays, though (e.g., Fig. 1). Temperature optima vary between taxa, with significant genotypic variation by latitude (Thomas et al. 2012), but growth rates frequently decline very rapidly at superoptimal temperatures: the median difference between the temperature optimum and the temperature at which growth was completely abolished in 163 cultures of phytoplankton was 7.1 °C (Cullen and MacIntyre 2016). In a similar vein, changes in growth irradiance should be tested prior to being imposed during grow-out to avoid exceeding genotypic and phenotypic limits on the irradiance at which photosynthesis and growth become light-saturated (MacIntyre et al. 2002). Imposition of significantly higher irradiances will result in photoinhibition of growth rather than more rapid growth (e.g., Kana and Glibert 1987).

Finally, the use of a more sensitive fluorometer affords a further reduction in *t*_end_ by a factor proportional to [ln(change in LLD_*F*_^−1^)] (Eq. 6, Table 3). This is illustrated in Fig. 10c, again for *Thalassiosira* and *Alexandrium*. The use of a customized in situ FIRe fluorometer (Satlantic, Halifax, NS, Canada) reduced LLD_Cell_ by approximately an order of magnitude, from 117 to 13 cells mL^−1^ for *Thalassiosira* and from 91 to 14 cells mL^−1^ for *Alexandrium*. This would result in further reductions in *t*_end_ of 1 and 4 days, respectively (Fig. 10c). As these factors are additive, the cumulative reductions for the two species were from 13 to 6 days for *Thalassiosira* and from 40 to 19 days for *Alexandrium*. The bulk of the reductions, 57 and 52%, respectively, can be attributed to optimization of growth rates, with an additional 29% reduction by using a 3-generation safety margin and 14–19% from using a more sensitive fluorometer.

The duration of the assay could also be reduced by increasing the number of viable cells per tube through preconcentration of the parent sample. While this does have the advantage of reducing the inherent sampling bias in the case of very low cell concentrations (Frazier et al. 2013), there is a risk of damaging cells during concentration, resulting in an underestimate of the number of viable cells. Again, pretesting is warranted before implementing the approach.

### Dose-response of UVC treatment

Adoption of the criterion of balanced growth prior to experimentation allowed definitive attribution of losses in viability to the experimental treatments (e.g., darkness ± UVC) rather than the experimental treatment plus confounding factors associated with growth conditions. The test of this assertion is repeatability, defined here as the agreement between independent assays conducted days or months apart. For our experiments, repeatability is verified by the observation that in all cases (repeated tests on each of 12 taxa), the results for replicate assays conducted days to months apart (different symbols in Fig. 5) were not statistically distinguishable from each other.

The repeatability of responses from cells in balanced growth provides a significant advantage beyond confidence in the results: the UVC dose-response curves could be used as benchmarks for future tests of viability assays without repeating the SDC-MPN analyses, if the same cultures were grown under the same conditions. This would be of considerable value when developing new assays for viability, as the SDC-MPN assays described here required repeated monitoring of more than 3000 individual cultures over a period of 2 ½ years.

Accepting that the dose-response curves presented here are accurate and that the criteria for test duration are appropriate requires an assumption that results are not significantly influenced by delayed repair of damage caused by UVC. Any recovery that occurred after the grow-outs were completed and had been scored would cause the SDC-MPN assay to underestimate the viability of the test populations. The first line of evidence that this does not occur is the observation that there was almost no effect of treatment on the growth rates of the treated samples by the time that growth could be observed in the grow-outs (i.e., when fluorescence had risen above LLD_*F*_). Because the primary target of UVC damage is the nucleotides (Sinha and Hader 2002; Cadet et al. 2014), persistent, nonlethal damage would be manifested as impaired protein synthesis and a subsequent reduction in growth rate. By inference, recovery of growth rate after treatment demonstrates that any nonlethal damage arising due to treatment must have been repaired before growth was observed. Analysis of the ratio of the fluorescence attributable to viable cells relative to viable cell concentration, ${F}_{\mathrm{Init}}^{\prime }$, indicates that the repair period was 1–2 days in the overwhelming majority of cases (Fig. 8b). This corresponds to a median lag of less than 1 generation, which is consistent with the rapid rate of repair of UVC damage observed in the chlorophyte *T. suecica* (Hull et al. 2017). It was significantly shorter than the median time for the growing populations to reach the LLD, so it would have been completed before growth was observable. Consecutive repair and then growth is consistent with prior observations of recovery from UV radiation, in which the time-scale of repair of cyclobutane pyrimidine dimers is hours (van de Poll et al. 2002; Rastogi et al. 2011), resulting in resumption of growth in 2 days or less (Takahashi et al. 2006).

There are very few published UVC dose-response curves for phytoplankton, and responses published for other taxa (Hijnen et al. 2006; Haji Malayeri et al. 2016) are not directly comparable with our data for two reasons. First, the dose dependencies presented here are proprietary so cannot be compared in absolute units. Second, most published curves are based on single-dose UVC exposure, in contrast to the regimen used here of UVC exposure/dark hold/UVC exposure. This was intended to simulate treatment of ballast water at intake and discharge of a 5-day voyage and by its nature is a dual-stressor treatment for a photoautotrophic organism. If representative, the dose-response curves for *Microcystis aeruginosa* (Sakai et al. 2011), *C. reinhardtii* (Chaudhari et al. 2015), and *T. suecica* (Olsen et al. 2015) indicate that phytoplankton are more resistant to UVC than the viruses, bacteria, and heterotrophic protists reviewed by Hijnen et al. (2006) and Haji Malayeri et al. (2016). However, the doses required to treat the species used in this study are within the ranges used to treat municipal wastewater (USEPA 2006; NWRI 2012).

A striking feature of the majority of dose-response curves presented here is the threshold below which UVC had no discernible effect on viability, often referred to as “shouldering” in the literature on disinfection (Weavers and Wickramanayake 2001; Hijnen et al. 2006). A minority of samples also had biphasic responses, often referred to as “tailing.” Our empirical model (Eq. 8) reproduces tailing as the combined effects of two targets with differing influences, represented by a partitioning coefficient, *α*, and respective sensitivities to UVC, *k*_1_ and *k*_2_ (mJ^−1^ cm). We used Eq. 8 to describe patterns in dose-response curves and to group them by species (Fig. 5, Table 4) without considering their mechanistic foundations.

A general model of disinfection was proposed by Jensen (2010), in which relative viability is described from the cumulative distribution function of a normal distribution. The model captures much of the variability observed in real-life responses, including both shouldering and tailing, but does so from a single damage point, rather than the two that are specified in Eq. 8. Although elegantly simple, the structure is almost certainly not representative of metabolic control, which is likely to be distributed through multiple components of a metabolic pathway (Kacser and Burns 1973). Application of Jensen’s model to our results is complicated by the fact that our study was based on application of two stresses, prolonged darkness without and with UVC. This makes it more unlikely that damage would correspond to a single structural target. Even so, the model presents a useful conceptual framework for considering the mechanisms that might underlie the observed responses.

A period of darkness is a predictable part of the day/night cycle and may be important for maintaining optimal growth rates (Brand and Guillard 1981). Nevertheless, extended darkness results in debilitation of cellular pathways as the demand for maintenance respiration is not balanced by autotrophic carbon fixation. While some mixotrophic phytoplankton are capable of heterotrophy in darkness (Martínez and Orús 1991; Manoharan et al. 1999; Lee 2001), in many others, mixotrophic carbon acquisition is light-dependent (Li et al. 1999; Walsby and Juttner 2006; Brutemark and Granéli 2011). Respiratory demands are therefore met by catabolic consumption of energy-storage compounds and structural components of the cells, resulting in degradation of photosynthetic competence (Yentsch and Reichert 1963; Popels et al. 2007; McKie-Krisberg et al. 2015). Ultimately, prolonged darkness can trigger programmed cell death (Franklin and Berges 2004; Segovia and Berges 2005). Statistically significant reductions in viability in the dark controls were observed in only 3 of the 9 species described here, accounting for mean reductions of 55–72%. This suggests that the stress imposed by darkness alone had not triggered mass mortality in the cultures.

Light is critical to recovery from UVC irradiation. The primary damage is from DNA lesions, notably cyclobutane pyrimidine dimers and pyrimidine 6-4 pyrimidone photoproducts, CPDs and 6-4PPs (reviewed by Sinha and Hader 2002; Rastogi et al. 2010a). Nucleotide repair is effected by multiple pathways and illumination is a direct component of one. Photorepair is catalyzed by light-sensitive flavoproteins, the cryptochrome/photolyase family, which are involved in light-sensing and signaling processes that both regulate protein synthesis and directly bind and repair DNA (reviewed by Oliveri et al. 2014; Lepetit and Dietzel 2015). There is also excision repair of damaged nucleotides (e.g., Rastogi et al. 2010a; Chaudhari et al. 2015). In contrast to photorepair, this is not light-dependent, so is often referred to as dark repair. There have been very few studies of photolyases in the phytoplankton; even so, it is clear that they differ in structure and location across taxa (Brunelle et al. 2007; Coesel et al. 2009; Heijde et al. 2010). Variability in rates of repair and the relative magnitudes of photorepair vs dark repair may underlie the wide ranges in *D*_Th_ and *k* observed here.

Irradiation with UVC results secondarily in the formation of reactive oxygen species that cause further damage, critically to the psbA (D1) protein in the PSII reaction center (Rastogi et al. 2010b; Wang et al. 2015). Photosynthesis provides the energy required for catabolic metabolism—including both repair and cell division—beyond what can be supported by internal energy-storage reserves. Sakai et al. (2011) measured viability following UVC irradiation in cultures of *Microcystis* with and without photorepair. A pronounced shoulder in the dose-response curve for cultures permitted photorepair and its absence in cultures without it, and the convergence of the curves at high doses suggests that loss of viability at low dose was primarily from nucleotide damage but the loss at high doses was primarily from another source. A dose-dependent transition between targets is implicit in Eq. 8b and may underlie the biphasic response observed in 5 of the 12 taxa studied.

Although ascribing mechanism to the wide differences in dose-response curves observed here can only be conjectural, it is clear that there are differences. The “best practice” approach used in this study, when coupled with maintenance of the cultures in balanced growth prior to experimentation, allowed demonstration that the measurements were consistent over time and between independent cultures. Beyond their utility in defining design specifications for shipboard treatment systems, the response curves are true indicators of viability—i.e., growth and reproduction (Breeuwer and Abee 2000)—following treatment. They could and should be used as benchmarks for testing other assays of viability, many of which are (strictly) assays of vitality (e.g., intact membranes, nucleotide functionality, and/or metabolic competence, Breeuwer and Abee 2000). The tests can be conducted by reproducing the growth conditions and UVC exposures for the tested strains—relative viability can be specified for each exposure without the need to repeat the SDC-MPN assay.

Given the recent ratification of the IMO’s Ballast Water Convention, and the need to assess treatment efficacy of ballast water treatment systems for certification and for shipboard compliance testing, there is a pressing need for rapid, reliable, and accurate assays. Candidates that have been proposed include the vital stains fluorescein diacetate and chloromethylfluorescein diacetate (FDA and CMFDA) required by US regulations (USEPA 2010) and variable fluorescence (e.g., First and Drake 2013, 2014). However, these assay membrane integrity and metabolic competence, respectively, rather than nucleotide functionality, which is the target for UVC damage. We have tested both against the SDC-MPN method for the 12 taxa described here and found that neither provides an accurate estimate of UVC-induced loss of viability (MacIntyre et al., unpublished).

## Conclusion

We argue that the appropriate metric for measuring invasive potential of organisms in ballast water is viability, the ability to reproduce, and not vitality, the manifestation of functional components of the cellular apparatus. Adaption of the Most Probable Number assay—a benchmark test used in microbiology—and development of appropriate “best practice” criteria for its application with phytoplankton allow the loss in viability following application of a biocide to be assessed repeatedly and consistently in laboratory experiments. When used to assay the responses of cells maintained in balanced growth under well-defined conditions, the SDC-MPN assay provides repeatable estimates of the dose-response function for UVC-based loss of viability. This is suitable for comparison with proxies of treatment effectiveness, even if experiments are conducted at different times.

## Abbreviations

IMO: International Maritime Organization
SDC-MPN: Serial-Dilution Culture-Most Probable Number
USCG: United States Coast Guard
UVC: Ultraviolet-C radiation
